# Immunoglobulin A Glycosylation Differs between Crohn’s Disease and Ulcerative Colitis

**DOI:** 10.1021/acs.jproteome.3c00260

**Published:** 2023-08-29

**Authors:** Florent Clerc, Karli R. Reiding, Noortje de Haan, Carolien A. M. Koeleman, Agnes L. Hipgrave Ederveen, Natalia Manetti, IBD-BIOM Consortium, Viktoria Dotz, Vito Annese, Manfred Wuhrer

**Affiliations:** † Center for Proteomics and Metabolomics, Leiden University Medical Center (LUMC), Postbus 9600, Leiden 2300 RC, The Netherlands; ‡ Biomolecular Mass Spectrometry and Proteomics, Bijvoet Center for Biomolecular Research and Utrecht Institute for Pharmaceutical Sciences, University of Utrecht, Padualaan 8, Utrecht 3584 CH, The Netherlands; § Netherlands Proteomics Center, Padualaan 8, Utrecht 3584 CH, The Netherlands; ∥ Unit of Gastroenterology SOD2 (Strutture Organizzative Dipartimentali), Azienda Ospedaliero Universitaria (AOU) Careggi, Florence 50134, Italy; ⊥ Gastroenterology Unit, San Jacopo Hospital, Pistoia 51100, Italy; # Unit of Gastroenterology, IRCCS (Istituto di Ricovero e Cura a Carattere Scientifico−Casa Sollievo della Sofferenza) Hospital, San Giovanni Rotondo 71013, Italy; ¶ Vita-Salute San Raffaele University Faculty of Medicine and Surgery, Milano 20132, Italy; ∇ IRCCS Policlinico San Danato, San Donato Milanese 20097, Italy

**Keywords:** inflammatory bowel disease, Crohn’s disease, ulcerative colitis, IgA1, glycosylation, biomarker

## Abstract

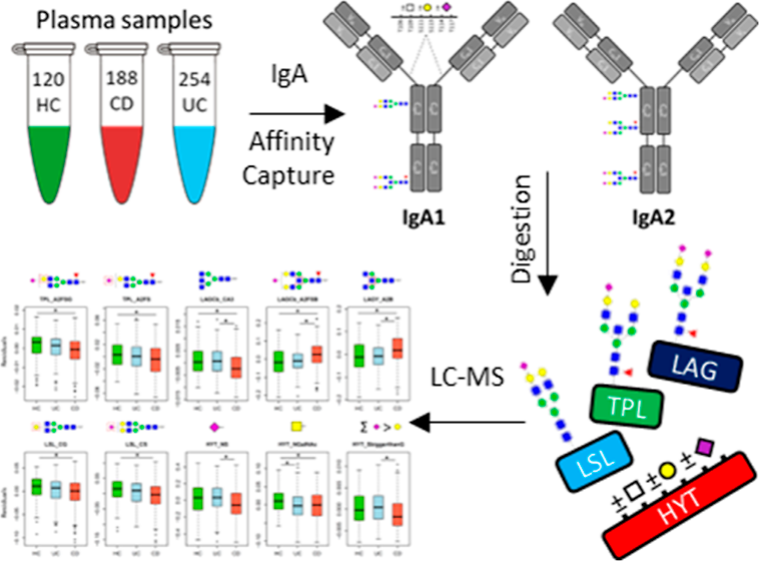

Inflammatory bowel diseases (IBD), such as Crohn’s disease (CD) and ulcerative colitis (UC), are chronic and relapsing inflammations of the digestive tract with increasing prevalence, yet they have unknown origins or cure. CD and UC have similar symptoms but respond differently to surgery and medication. Current diagnostic tools often involve invasive procedures, while laboratory markers for patient stratification are lacking. Large glycomic studies of immunoglobulin G and total plasma glycosylation have shown biomarker potential in IBD and could help determine disease mechanisms and therapeutic treatment choice. Hitherto, the glycosylation signatures of plasma immunoglobulin A, an important immunoglobulin secreted into the intestinal mucin, have remained undetermined in the context of IBD. Our study investigated the associations of immunoglobulin A1 and A2 glycosylation with IBD in 442 IBD cases (188 CD and 254 UC) and 120 healthy controls by reversed-phase liquid chromatography electrospray-ionization mass spectrometry of tryptic glycopeptides. Differences of IgA *O-* and *N*-glycosylation (including galactosylation, bisection, sialylation, and antennarity) between patient groups were associated with the diseases, and these findings led to the construction of a statistical model to predict the disease group of the patients without the need of invasive procedures. This study expands the current knowledge about CD and UC and could help in the development of noninvasive biomarkers and better patient care.

## Introduction

Interest in large glycomics studies is rapidly increasing and associations of plasma protein glycosylation with disease have been demonstrated for cancers, autoimmune diseases, and inflammatory bowel disease (IBD).^1–5^

IBD, comprising Crohn’s disease (CD) and ulcerative colitis (UC) are lifelong, chronic, and include relapsing inflammatory conditions of the digestive tract with yet unknown etiology.^6^ CD and UC have similar symptoms but respond differently to medication and surgical treatments and the distinction between the two often requires invasive procedures that might include intestinal tissue sampling.^7^ Early and noninvasive patient stratification from readily available body fluids is needed to best target therapy.

Immunoglobulin G (IgG) is of particular interest as an inflammatory marker in IBD because it is widely involved in autoimmunity. Having established the biomarker potential of the IgG glycosylation and total plasma *N*-glycome (TPNG) in IBD, IgA1 seemed to be an ideal additional candidate to receive attention due to its presence in mucosal fluids of the intestinal tract and its established role in immunological responses and mucosal protection against microorganisms such as bacteria and viruses. The latter happens by binding or agglutinating them, thus promoting clearance and preventing infections and inflammations.^3,4,8–12^ However, the function of IgA glycosylation in health and disease is still an active area of research.

The *O*- and *N*-glycosylation of IgA1 and IgA2 have previously been monitored for 29 women during pregnancy and in the postpartum period using matrix-assisted laser desorption/ionization-Fourier-transform ion-cyclotron resonance (MALDI-FTICR).^13^ The same MALDI-FTICR method has again been used for the analysis of plasma IgA from 284 individuals comprising both rheumatoid arthritis (RA) patients and controls.^14^ However, the complex and technically challenging sample handling used in these studies is an obstacle in large-scale clinical studies. Recently, an alternative LC–MS(/MS) approach with reduced benchwork sample preparation and a more streamlined workflow has been successfully applied to analyze IgA glycopeptides in a cohort of IgA nephropathy patients and healthy controls.^15^ The same method was selected for this research.

Next to its important functions in intestine immunity, IgA was also implicated as a potential target glycoprotein based on previous TPNG analysis. Specifically, the IgA glycans expectedly contribute to a large extent to specific plasma glycoforms affected by IBD, including elevated bisection levels of the diantennary fucosylated sialylated glycans.^4,16^ IgA1 and IgA2 glycoforms have previously been associated with CD, UC, and IgA nephropathy (IgAN) where the general glycan size was reduced (lower levels of sialylation, galactosylation and antennarity of *N-*glycans, and reduced sialylation and galactosylation of *O*-glycans) as well as with secondary IgA nephropathy where nephropathy follows CD, indicating the potential involvement of IgA in these diseases.^15,17–21^ Small-scale studies have also demonstrated changes in the *O*-glycosylation traits of IgA such as reduced levels of GalNAc in IBD.^22,23^ Still, the overall role of IgA glycosylation in the disease remains largely unclear. It is mostly unexplored to which extent IgA glycosylation affects FcαR receptor binding triggering pro- or anti-inflammatory responses, or clearance from the circulation by the asialoglycoprotein receptor.^16,24,25^

In this study, the IgA glycosylation profiles of 442 IBD patients and 120 healthy controls were analyzed by LC–MS/MS with glycosylation site-specificity. The main goal was to find associations between IgA glycosylation and IBD characteristics to discriminate CD, UC, and HC, and subsequently, in a secondary analysis, to find associations with disease behavior and surgical and medical treatments for improved patient care.

## Materials and Methods

### Clinical Samples

The plasma samples used for this analysis were received from the University Hospital Careggi (Florence, Italy) and consisted of 442 IBD cases (188 CD and 254 UC) and 120 healthy controls (HC) as previously described.^4,26^ Written informed consent was obtained from the study participants, and the ethical approval was obtained from the Ethics Review Board of the Casa Sollievo della Sofferenza Hospital (Italy). The clinical information on CD and UC patients was collected following the rules of the Montreal classification of inflammatory bowel disease.^27^ The demographics of the individuals included in the statistical analysis are detailed in Table 1.

**Table 1:** Demographics of the Individuals Included for Statistical Analysis

sample numbers	HC	CD	UC
total (males)	120 (72)	188 (110)	254 (159)
Age, mean ± SD			
males	48.6 ± 16.1	36.0 ± 13.6	42.2 ± 16.1
females	60.0 ± 17.8	37.3 ± 15.6	42.1 ± 16.7
all	53.2 ± 17.7	36.5 ± 14.4	42.1 ± 16.3

patient information		
disease group (counts (males))	CD	UC
Disease Location (CD)		
illeum± upper GI* (L1 ± L4)	65 (39) ± 1 (1)	
colon ± upper GI (L2 ± L4)	67 (44) ± 6 (4)	
illeum+ colon ± upper GI (L3 ± L4)	47 (20) ± 1 (1)	
upper GI (L4)	8 (6)	
Disease Location (UC)		
rectum (E1)		29 (15)
sigmoid + left colon (E2)		127 (79)
entire colon (E3)		93 (61)
Disease behavior (CD)		
inflammatory (B1)	106 (64)	
stenosing (B2)	55 (27)	
fistulizing (B3)	26 (18)	
Surgery		
no	127 (74)	243 (151)
before sample collection	49 (28)	8 (5)
after sample collection	8 (5)	2 (2)
Drugs		
mesalazine	38 (19)	75 (46)
steroids	42 (25)	79 (49)
azathioprine/6-mercaptopurine	43 (26)	58 (38)
anti-TNFα	51 (31)	22 (14)
*GI = gastro-intestinal (tract)		

### Glycoproteomics Analysis

#### Capture of IgA

IgA1 and IgA2 were captured from five μL of plasma (containing approximately 350 μg of total protein and 13 μg of IgA) with camelid antibody domains immobilized on agarose beads (CaptureSelect IgA Affinity Matrix, Thermo Fisher Scientific, Waltham, MA) as previously described.^13^ Ten μL of bead slurry were pipetted into each well of a 96-well Orochem filter plate (AcroPrep, Pall Corporation, Ann Arbor, MI, USA). The beads were washed three times with 200 μL ultrapure water (prepared at 18.2 MΩ with a Purelab Ultra, Veolia Water Technologies Netherlands B.V., Ede, The Netherlands). and three times with PBS 1× solution on vacuum manifold. Five μL of sample were added to 100 μL of PBS in each well and incubated with shaking for 1 h at room temperature (RT). The beads were washed on vacuum manifold with 3 × 200 μL PBS 1× and three times 200 μL water. The captured IgA was released from the beads with 100 μL of 100 mM formic acid, eluted in PP V-bottom plates by centrifugation (1 min, 100*g*), and dried at 50 °C in a vacuum centrifuge.

#### Trypsin Digestion

The samples were reconstituted in 100 μL of 20 mM ammonium bicarbonate buffer and reduced with 2 μL of dithiothreitol (DTT) 125 mM for 30 min at 60 °C. 4 μL of 125 mM of iodoacetamide (IAA) was added for alkylation, and the samples were incubated with shaking for 30 min at RT wrapped in aluminum foil. The alkylation was quenched by adding 2 μL of 100 mM DTT. The digestion was performed overnight at 37 °C with 210 ng of TPCK-treated trypsin, and the samples were frozen at −20 °C until measurement.

#### RP-LC-ESI-MS(/MS)

Reverse-phase liquid chromatography electrospray-ionization mass spectrometry (RP-LC-ESI-MS) experiments were performed with an Ultimate 3000 RSLCnano system (Thermo Fisher Scientific) fitted with a Pepmap100 C18, 5 μm 0.3 × 5 mm precolumn and a custom-made Acquity BEH130 C18 75 μm x 100 mm UPLC M-Class analytical column. This was coupled to a Maxis Impact HD quadrupole-time-of-flight (qTOF) mass spectrometer fitted with a captive spray nanoBooster (Capillary voltage: 1200 V; nanoBooster pressure, 0.2 bar; dry gas flow: 3.0 L/min; dry gas temperature: 180 °C) (Bruker Daltonics, Bremen, Germany). One μL of digested sample was injected, with a flow rate of 500 μL/min, eluted with the following gradient: 3% solvent B at 0 min, 30% B at 6.5 min, 95% B at 10 min, hold for two min, back to 3% B at 13 min, hold for eight min. Solvent A consisted of 0.1% TFA (v/v), solvent B consisted of 95% ACN (v/v)). Mass spectra were recorded from 550 to 1800 Th at a frequency of 1 Hz.

To confirm the structures of certain analytes, high-resolution fragmentation spectra of selected glycopeptides were recorded on a different instrument. The material used for the MS/MS glycopeptide identification was a pooled sample of captured IgA taken from the first 96-well plate of the prepared samples including material from HC, CD, UC, and control plasma (VisuconF, Affinity Biologicals, Canada). For this, a nanoLC (Thermo Fisher Scientific) was equipped with an Acclaim PepMap 100 trap column (100 μm × 20 mm, particle size 5 μm, Thermo Fisher Scientific) and an Acclaim PepMap RSLC C18 nanocolumn (75 μm × 150 mm, particle size 2 μm, Thermo Fisher Scientific). The separation gradient started from 97% solvent A (0.1% formic acid in water) and 3% solvent B (95% ACN) to 53% solvent B over 30 min with a flow rate of 700 nL/min. The system was coupled to a maXis quadrupole time-of-flight MS (q-TOF-MS; Bruker Daltonics) equipped with a nanoBooster (Bruker Daltonics). Ionization was enhanced by applying acetonitrile-enriched gas at 0.2 bar, which was used as a dopant. The source parameters were set to a flow of nitrogen drying gas of 3.0 L/min at 180 °C and a capillary voltage of 1200 V. The MS was operated in stepping-energy CID mode and acquired from *m*/*z* 50–2800 with the precursor selection above *m*/*z* value 680 as previously described.^28^

#### Data Processing

Raw LC–MS data were converted to mzXML with MSConvert. LacyTools^29^ was used to align the retention time, calibrate the mass spectra, define time windows around each glycopeptide cluster extraction, and for the integration of the sum spectra for a list of analytes determined by manual exploration of the data of each disease group using sum spectra and theoretical glycosylation sites based on the peptide sequence (Uniprot #P01876 for IgA1 and P01877 for IgA2). For time alignment, the most abundant glycopeptide of each cluster was used (search windows of 20 s), and the maximum mass error of the highest isotope was ±0.15 Th. A time window of ±8 s per cluster (10 s for the *O*-glycopeptide) was summed, and each extracted glycoform was reported as relative percentage per cluster. The optimal time windows for analyte extraction were determined for each cluster by monitoring the elution profile and retention time of all extracted analytes per glycopeptide cluster. The average retention time of the cluster was then calculated and the sum windows fixed to include all analytes. The extracted glycopeptide data was curated on average signal-to-noise ratio threshold >9, absolute error on exact mass <20 ppm, and deviation compared to the calculated isotopic pattern <0.2. This curation was applied for all detected charge states. Per sample, the data was kept when not more than one peptide cluster was rejected due to low-abundance of analytes. A final list of 51 glycopeptides was retained (26 *N*-linked and 25 *O*-linked glycopeptides (Tables S1 and S2), and from these, 45 derived traits were calculated (Table S3).

The identity of glycopeptides was verified by MS/MS using Byonic. The search parameters included the protein sequences of secreted IgA1 (P01876-1) and IgA2 (P01877-1) from Uniprot in FASTA format, cysteine alkylation as fixed peptide modification, two possible missed cleavages, and a semispecific (N-ragged) digestion specificity. Furthermore, a list of 318 N-glycan compositions and 9 O-glycan compositions was included based on IgA glycoforms previously identified (based on monosaccharides HexNAc, Hex, Fuc, NeuAc, and NeuGc).^15,30,31^ The precursor mass tolerance was set at 10 ppm, and the fragment mass tolerance at 30 ppm. The results of the Byonic search were filtered based on the identification score (Pep2D) to be below 0.001, manually expected (at least one glycoform per peptide portion) and are reported in Table S2 and Figures S1–S4. The identity of the IgA glycoforms that were not reported by Byonic was deduced by exact mass differences in the high-resolution MS1 data, including retention time and isotope pattern matching (Figures S5–S9).

#### Statistical Analysis

The associations of the IgA glycopeptides and calculated derived traits with CD, UC, and IBD-specific clinical traits, i.e., extent of the disease, drug intake, and surgical intervention, were tested as previously described in the software R studio for R (Version 1.0.136).^4^ In short, the data were batch corrected (on logarithmically transformed data) to account for the different preparation days, position on row, and column of the randomized samples on the 96-well plates. Associations with age and sex were determined in the three patient groups with linear and logistic regressions and included further as covariates in the logistic regression model. The associations of the IgA glycosylation with age, sex, Crohn’s disease, ulcerative colitis, and IBD-related parameters such as disease location, surgery, and medication were tested by linear and logistic regression on scaled data (subtraction of the mean and division by the SD). Age, sex, and their interaction were included as covariates in the models for the disease-related tests. The odds ratios (OR) were calculated with their 95% confidence intervals (CI) assuming a Student’s t-distribution and are, thus, representative of single standard deviation increases in the tested traits.

For the receiver operating characteristic (ROC) analysis, the glycopeptides most significantly associated with the IBD (*p-*values and effect sizes) were selected for the initial model. The ones found to not significantly contribute to the model were removed step-by-step considering similarities of derived traits (which can share effects in the model and lower their individual significance while only reflecting a single biological trait) and percentage of effect on the prediction until only significant traits were left, thus achieving maximal prediction power with only the necessary selection of traits. The model was trained 20 times on a random selection of 75% of the samples and the prediction was calculated for the respective 25% remaining. The standard error (SE) was also calculated for all predictions.

The Montreal classification of inflammatory bowel disease was followed to categorize disease localization, extent and behavior for CD and UC patients.^27^ The different localization and extent of the disease were compared in CD patients affected either in the ileum (L1), the colon (L2), or both the ileocolon (L3) and in the upper gastrointestinal (GI) tract (L4) (L1 vs L2 vs L3+L4). Association with disease behavior was tested by comparing the inflammatory (B1), the stenosing (B2), and the fistulizing variants (B3) (B1 vs B2 vs B3). In UC, the patient groups affected in the rectum + sigmoid + left colon (E1+E2) were compared to the ones affected in the entire colon (E3), (E1+E2 vs E3). The hypothesis tested here is that a more extensive or aggressive disease behavior would associate with more extreme glycosylation profiles compared to HC.

We also tested the association of profiles with surgery and medication. According to the guidelines of the respective countries to treat IBD diseases, patients were given mesalazine, steroids, azathioprine/6-mercaptopurine, or anti-TNFα. Patients were stratified according to escalation of drug class used at the time of sample collection according to the practices of the medical center and the glycosylation profiles respective to each drug class were compared to each other.^32,33^ The hypothesis tested here is that patients suffering from more severe disease exhibit more extreme IgA glycosylation profiles and these patients can be identified based on their need to use more potent drugs. The groups of patients not requiring surgery were opposed to those who received surgery after sample collection, again using surgical intervention as a proxy for the disease severity. Moreover, we compared the profiles of patients who received surgery before or after sample collection to evaluate the treatment effect.

The commonly accepted significance threshold of α = 0.05 was adjusted for multiple testing with a Bonferroni correction when evaluating the associations with age, sex, CD, and UC. As the relative intensities of the glycopeptide signals are intimately related, the number of tests performed on the derived traits was used as a correction factor.

## Results

**Figure 1:**
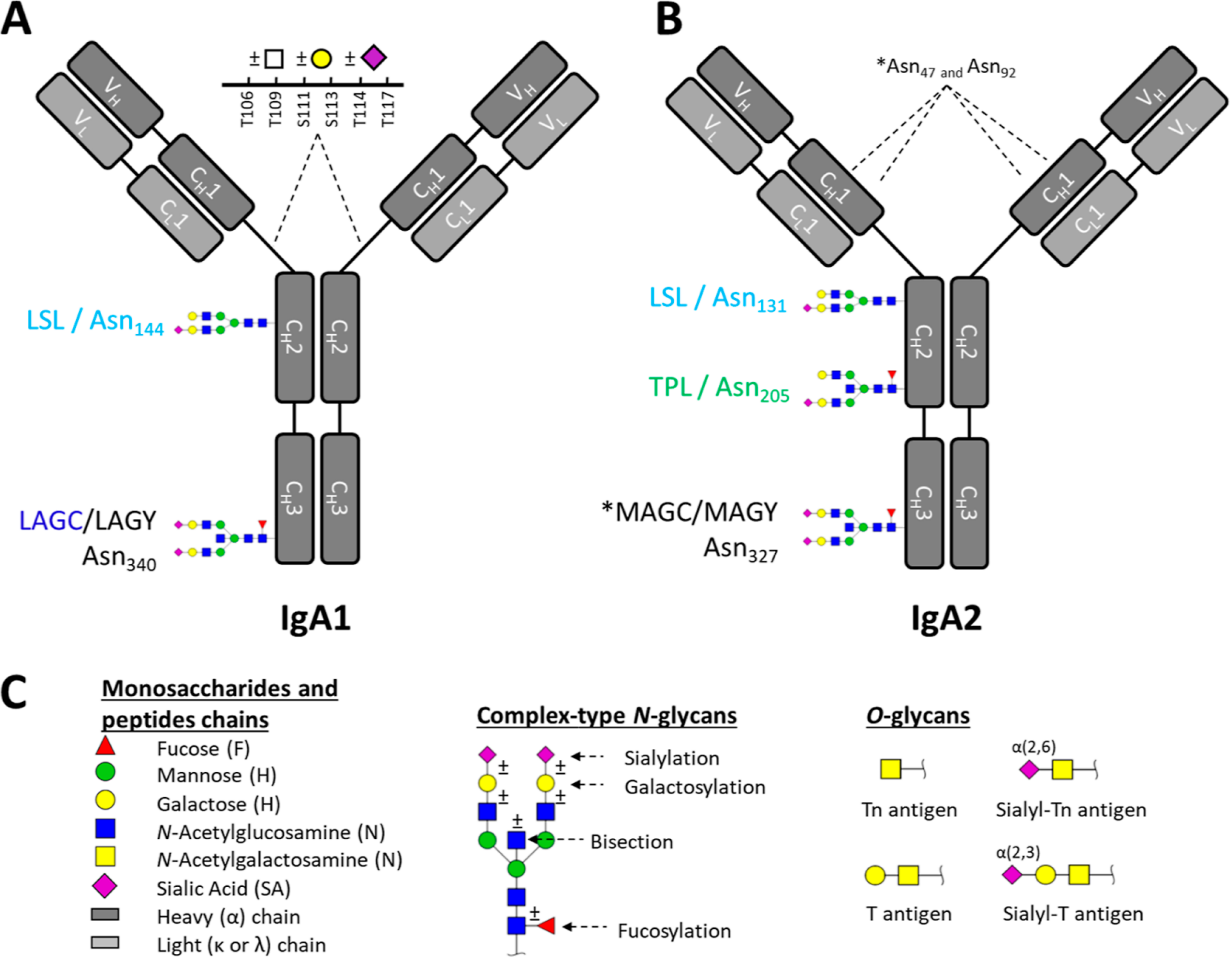
Symbolic representation of IgA1 and IgA2 glycosylation. The acronyms HYT, LSL, TPL, LAG(C/Y), and MAG(C/Y), which refer to the different glycopeptides, come from the first three amino acids of the corresponding tryptic digest sequence of IgA1 and IgA2. (A) IgA1 has two *N*-glycosylation sites at Asn_144_ and Asn_340_ that are occupied by complex-type N-glycans. IgA1 also contains six O-glycosylation sites in the hinge region at Thr_106_, Thr_109_, Ser_111_, Ser_113_, Thr_114_, and Thr_117_. The O-glycosylation of IgA1 is represented as one combined monosaccharide composition, as no information about specific glycan structures and attachment sites was obtained. (B) The observed glycosylation sites of IgA2 at Asn_131_, Asn_205_, and Asn_327_ are occupied with complex-type *N*-glycans. * The IgA2 glycosylation sites Asn_47_ and Asn_92_ and Asn_327_ did not pass quality curation criteria due the low intensity of analytes. (C) Symbols used for representing the glycans and their general antennary structure (N-linked glycans) and example structures for the *O*-linked glycans.

**Figure 2:**
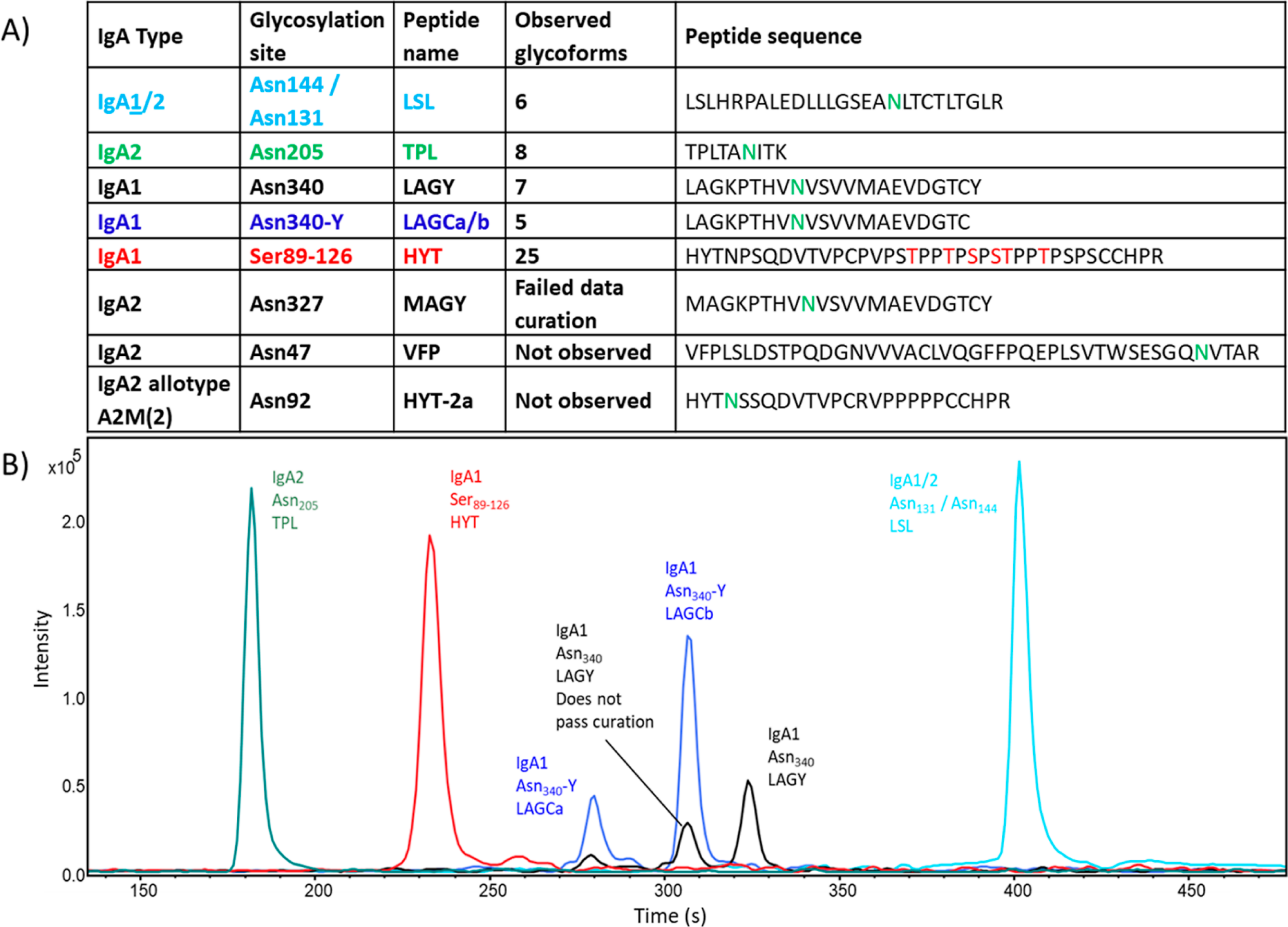
Description of the glycopeptide clusters. (A) IgA1 and IgA2 glycopeptides overview. Glycosylation sites, short peptide names, number of extracted glycoforms, and peptide sequences are given for each glycopeptide cluster. (B) Extracted Ion Chromatograms (EIC) of one selected glycopeptide per cluster with representative retention time of the whole glycopeptide cluster.

Following site-specific IgA1/IgA2 glycosylation analysis by LC–MS/MS, a total of six *N*-linked glycoforms was characterized on Asn_144_, eight on Asn_205_, seven on Asn_340_, five on the two different clusters of the truncated peptide containing Asn_340_, and 25 *O*-linked glycoforms on the peptide portion containing Ser_89–126_ (Figures 1, 2). Some glycopeptides similar to those found on Asn_340_ of IgA1 were observed on Asn_327_ of IgA2 (10× less abundant than IgA1) during the manual exploration of the data, but the list of potential analytes did not pass the curation step during data extraction due to a low S/N ratio, also impacting their isotopic pattern match compared to the calculated pattern so they were rejected from the statistical analysis. No confident identifications for the IgA2 glycopeptides covering Asn_327_ were found starting with amino acids MAG instead of LAG (IgA2 isoform #P01877-1) in the Byonic workflow. No glycopeptides passing our quality criteria were observed for the theoretical glycosylation site of Asn_47_ of IgA2 nor for Asn_92_ of the low-abundance allotype A2M of IgA2. Trace amounts of the what could be VFP glycopeptide (Asn_47_) fragments with a different cleavage at the N-terminal of its sequence (SES) were detected but not further evaluated due to their low abundance. For clarity of the discussion, the peptides will be referred to by the one-letter codes of the first three amino acids (and last for the peptides with a truncated variant); amino acid sequences are shown in Figure 2.

The identification of the glycoforms of the different detected peptides is depicted in Figures 3, 4 and in the Figures S1–S9. The peptide portion was annotated based on MS/MS data of high abundant parent ions as shown in Table S1.

**Figure 3:**
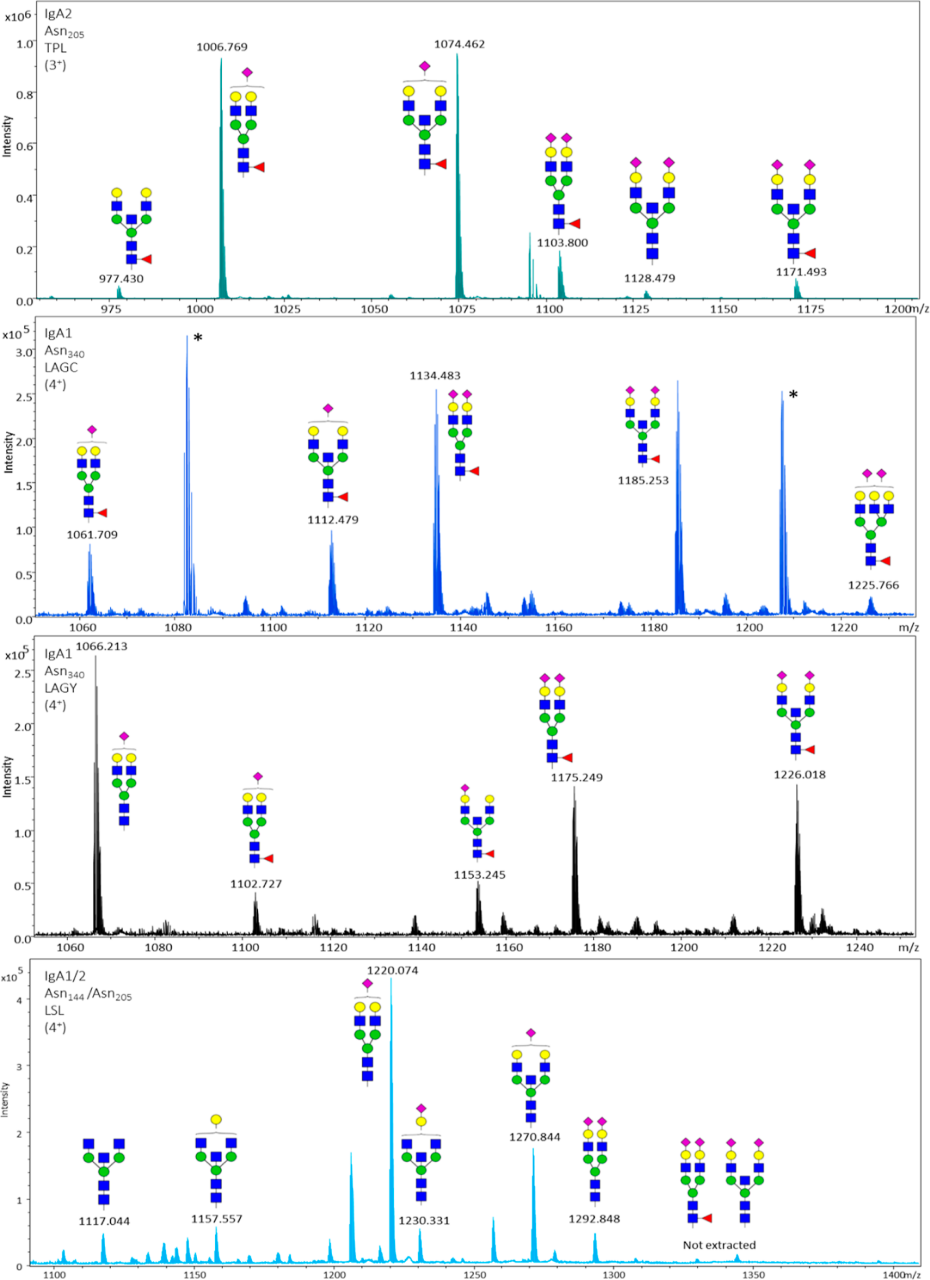
Assignment of the major N-glycoforms of IgA1 and IgA2. (A) Peptide cluster TPL at Asn_205_ of IgA2, zoomed-in view of the 3^+^ charged glycopeptides. (B) Truncated peptide cluster LAGC at Asn_340_ of IgA1, zoom-in of the 4^+^ charged glycopeptides. The two peaks marked with an asterisk are residues from another peptide without glycan eluting at the same time and in the charge state 2^+^. (C) Peptide cluster LAGY at Asn_340_ of IgA1, zoom-in of the 4^+^ charged glycopeptides. (D) Peptide cluster LSL at Asn_144_/Asn_205_ of IgA1 and IgA2 and zoom-in of the 4^+^ charged glycopeptides. All of the glycoforms are described in Table S1.

**Figure 4:**
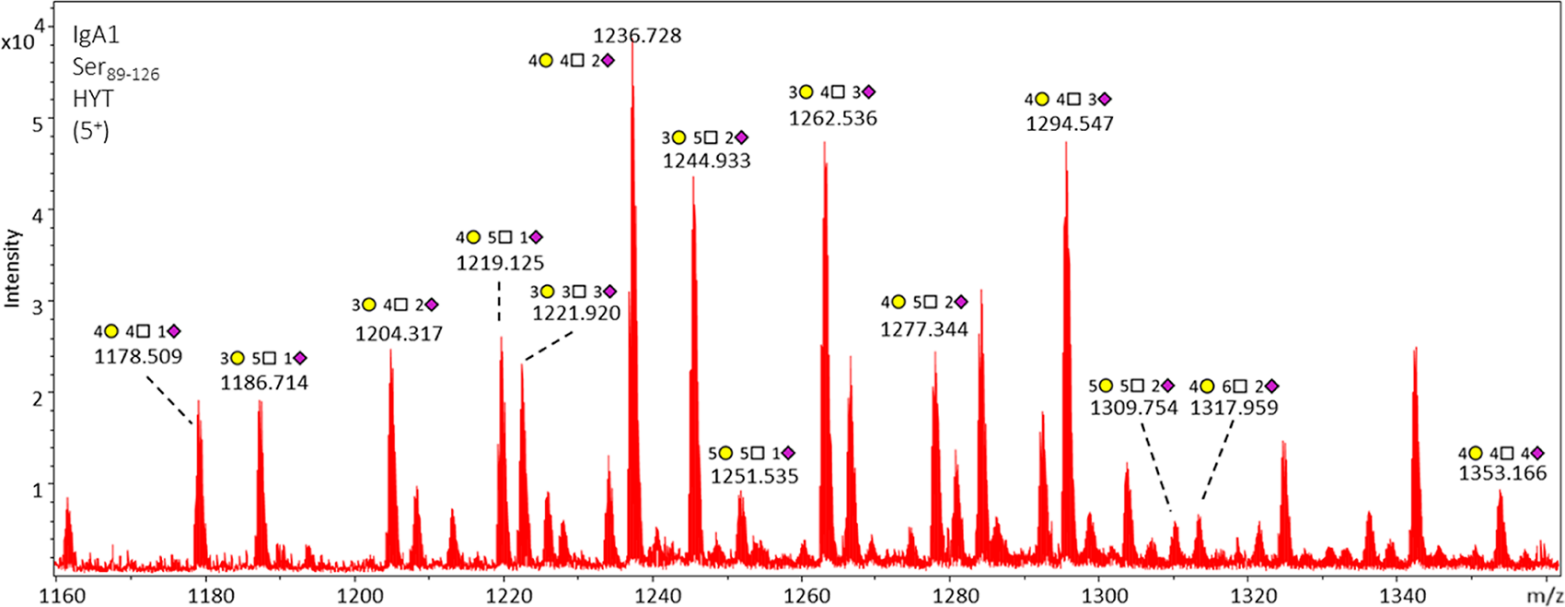
Assignment of O-glycoforms of peptide cluster HYT. Assignment of *O*-glycoforms of peptide cluster HYT at Ser_89–126_ of IgA1 and zoom-in of the 5^+^ charged glycopeptides. The pictograms represent the combined *O*-glycopeptide monosaccharide composition from all sites and do not convey information on specific glycan structures of attachment sites. Overall, the glycopeptides covered a wide range of compositions (H_3_–_5_N_3_–_6_S_1–5_) containing up to 6 N. Not all glycoforms are labeled on the figure, as the complete list of the 25 IgA1 hinge region *O*-glycopeptide species identified and quantified is reported in Table S1.

### IgA Glycosylation is Associated with Age and Sex in Healthy Controls and IBD Patients

In HC, CD, and UC, an increase of bisection and a decrease of galactosylation and sialylation per galactose were observed with age on all *N*-glycosylation sites (TPL, LAGY, and the truncated LAGC glycopeptides) as reported in Table S4. A decrease of antennarity was observed in the glycans of the TPL peptide of HC and on the glycans of the LAGY peptide of HC and UC patients. No association between the *O*-glycans of the HYT peptide and age was found to be significant after correction for multiple testing.

The *N*-glycosylation of IgA was associated with sex, where a decrease of antennarity was observed on the TPL peptide in CD and UC males compared to the females; Table S4. UC males had lower bisection levels than the females in all of the *N*-glycopeptides clusters.

### IgA Glycosylation is Associated with Inflammatory Bowel Disease

**Figure 5:**
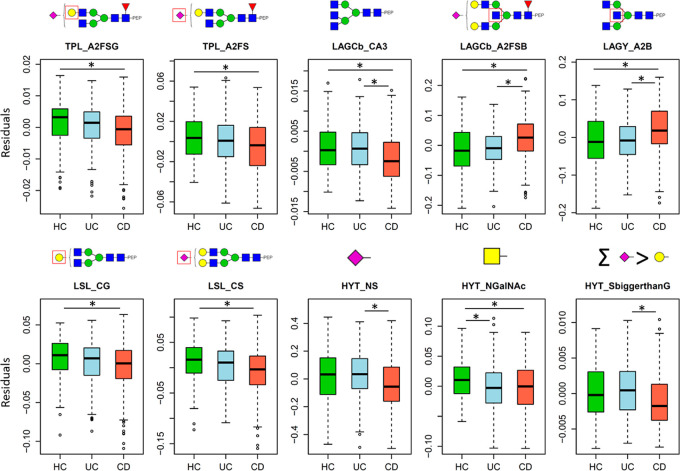
Main associations between IgA glycosylation-derived traits and IBD per patient groups (HC = healthy controls, UC = ulcerative colitis, and CD = Crohn’s disease). The derived traits were corrected age, sex, and age*sex and are shown in boxplots. The 25th, 50th, and 75th percentiles are represented in the boxes, and the whiskers are at the 1st quartile −1.5 × IQR (interquartile range) and at the 3rd quartile +1.5 * IQR. *P*-values, ORs, and confidence intervals are reported in Table S4. Statistically significant associations (multiple testing corrected threshold 3.70 × 10^–4^ as described in the supplementary tables) are marked with an asterisk.

Compared to HC, CD patients were found to have lower galactosylation and lower sialylation on LSL (Asn_144_) and TPL (Asn_205_) (Figure 5 and Table S5). CD patients also showed lower levels of antennarity (lower CA3) and higher bisection on Asn_340_ and its truncated versions compared to those of HC and UC. CD and UC patients had a smaller amount of GalNAc on the *O*-glycans of the Ser_89–126_-containing peptide than HC. In addition, the CD group was found to have a lower relative abundance of SA and a lower ratio of SA per galactose in the *O*-linked glycans than in UC.

**Figure 6:**
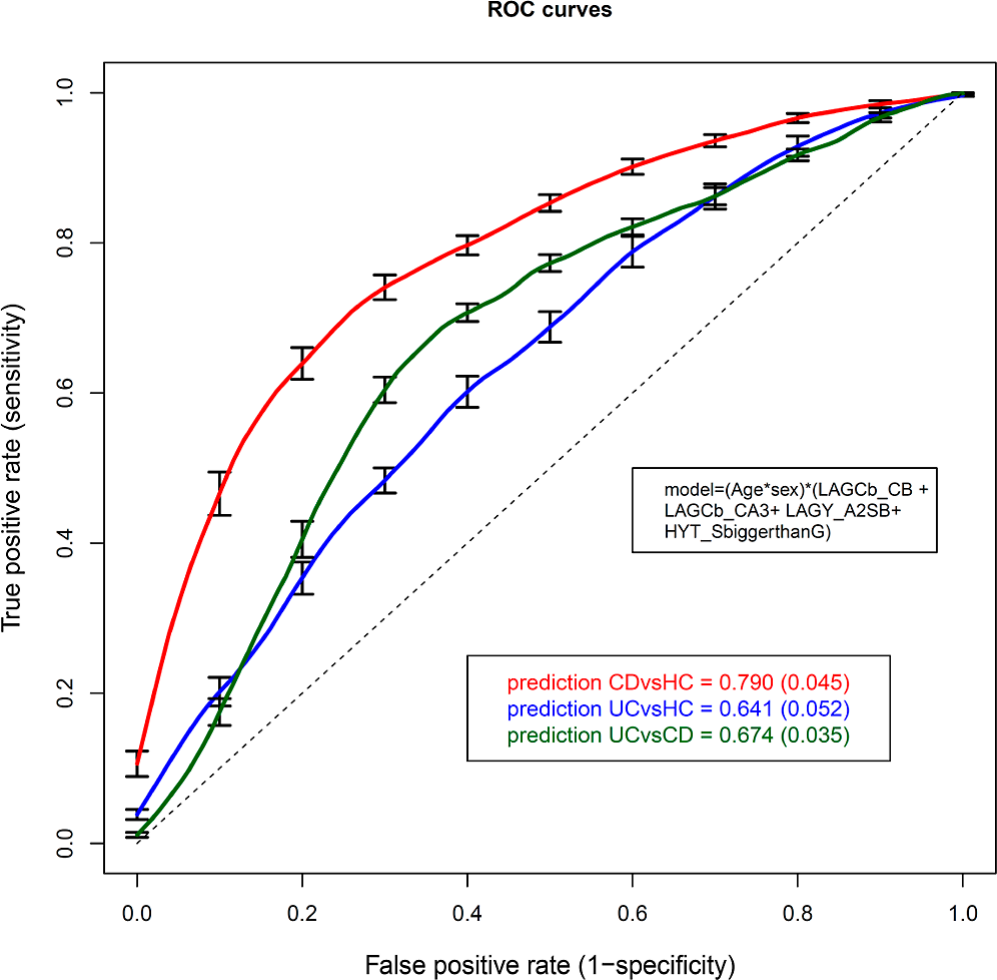
Receiver operating characteristic curves showing the power of selected glycan traits to predict CD and UC. The mean and SE of 20 predictions are reported for the area under the curve. The prediction model included the interactions of age*sex *(LAGCb_CB + LAGCb_CA3 + LAGY_A2SB + HYT_S > G).

The ROC curves (Figure 6) that were calculated with a linear model based on the glycopeptides most significantly associated with IBD included age, sex, and their interaction with the bisection and triantennarity of LAGC (LAGCb_CB, LAGCb_CA3), the bisection of sialylated diantennary LAGY glycopeptides (LAGY_A2SB), and the sum of *O*-glycopeptides featuring higher number of SAs than galactoses (HYT_S > G). The prediction power for CD vs HC was good with an AUC of 0.790 ± 0.045. The power of the model was fair for predicting UC vs HC with an area under the curve (AUC) of 0.641 ± 0.052 SE and also fair for UC vs CD with an AUC of 0.674 ± 0.035.

Associations of IgA glycopeptides with CD and UC disease location and behavior were tested as previously described^3,4^ but no significant results were found in this study (Tables S6–S8). No significant associations were found between patients who received surgery vs nonsurgery patients, and no associations were found in IgA glycosylation profiles of patients before and after surgery (Table S9).

**Figure 7:**
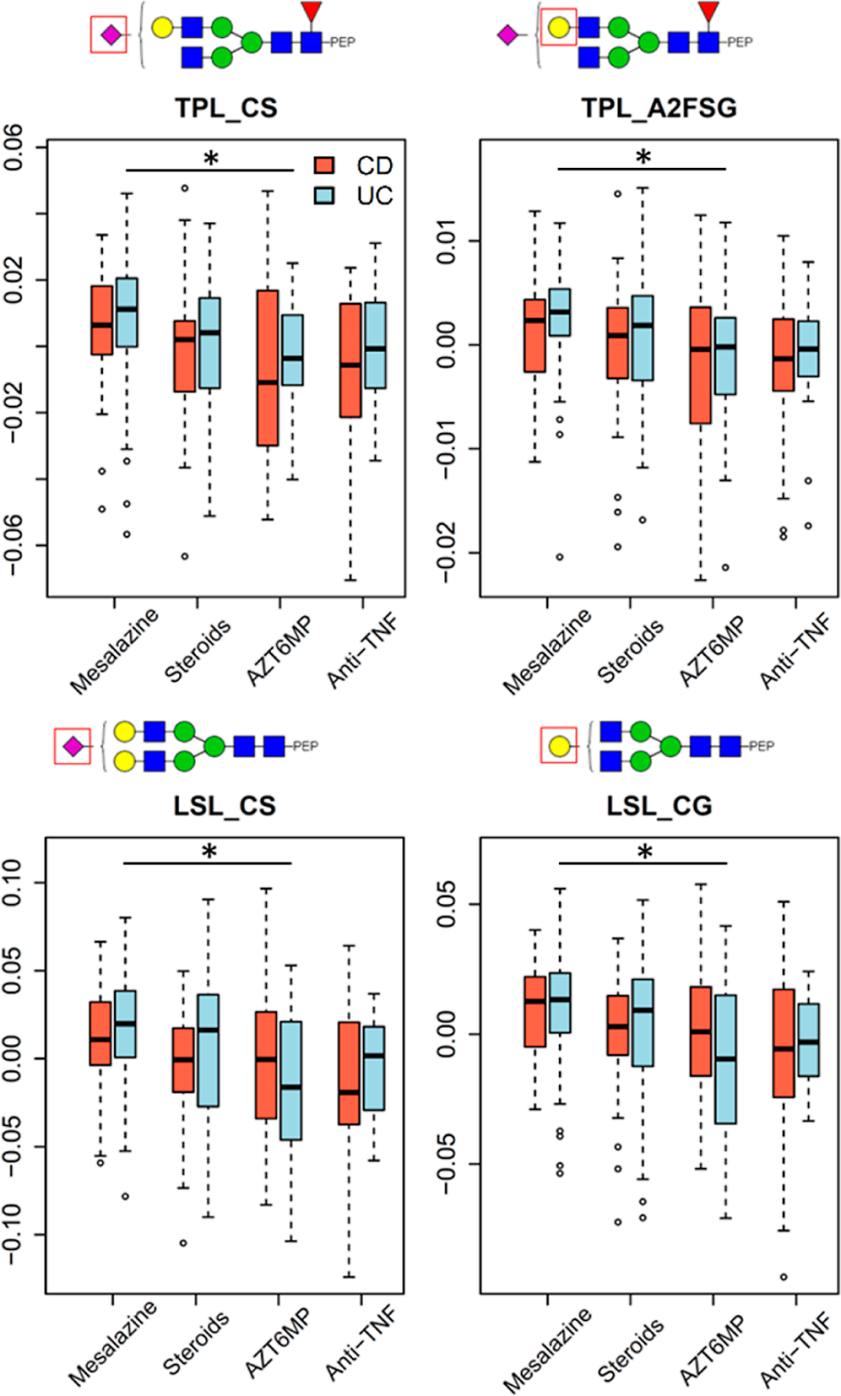
Main associations between IgA glycosylation derived traits and medication in CD and UC. The derived traits were corrected age, sex, and age*sex and are shown in boxplots. The 25th, 50th, and 75th percentiles are represented in the boxes and the whiskers are at the 1st quartile −1.5 * IQR (interquartile range) and at the 3rd quartile +1.5 * IQR. P-values, ORs, and confidence intervals are reported in Table S9. Statistically significant associations (multiple testing corrected threshold 1.85 × 10^–4^ as described in the Supplementary Tables) are marked with an asterisk.

When searching for associations of IgA glycosylation with the most potent drug administered to the patients, UC patients using AZT/6-MP were found to have lower galactosylation and lower sialylation of TPL and LSL peptides compared with UC mesalazine users (Figure 7).

## Discussion

**Figure 8:**
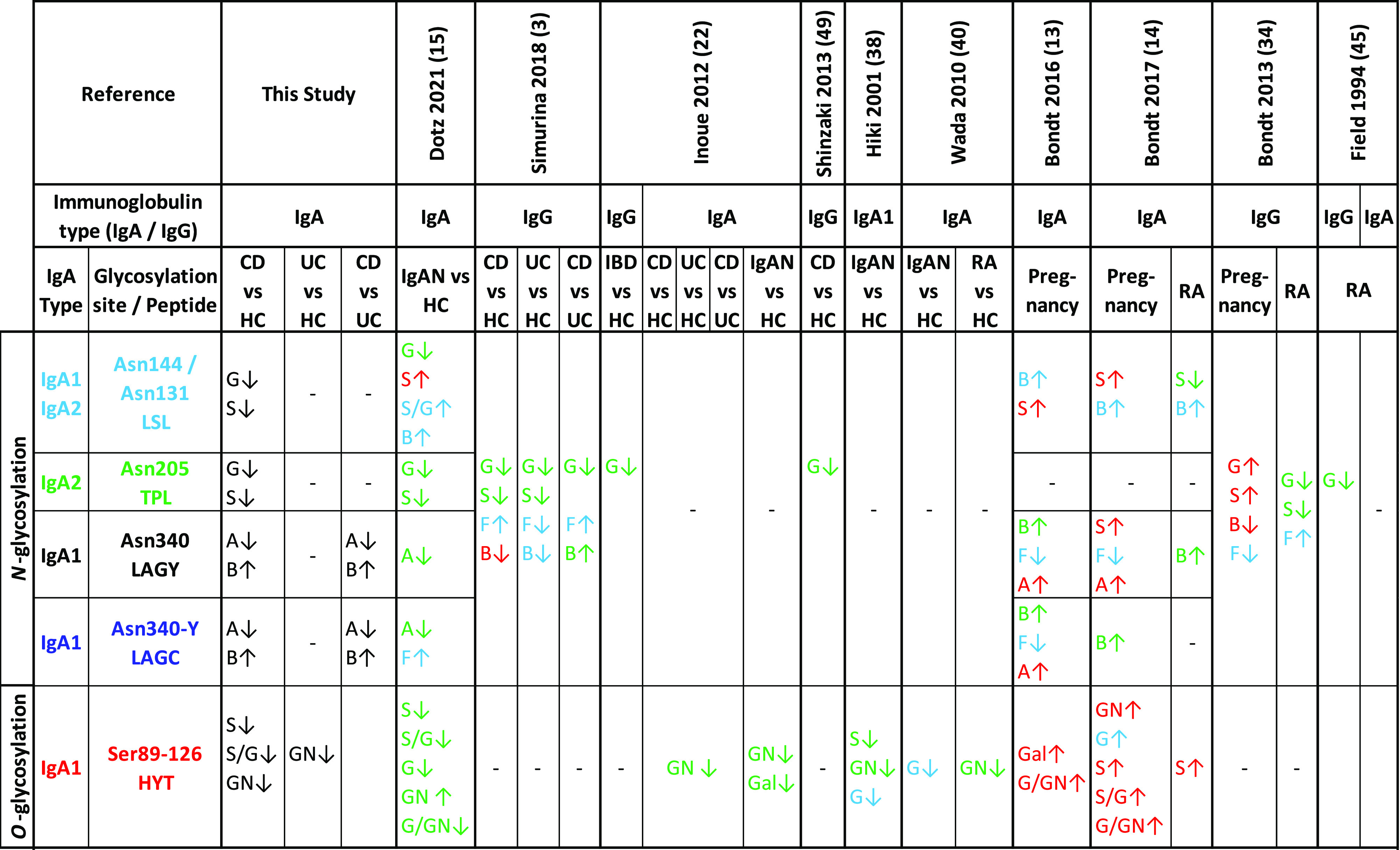
Comparison of associations found between IgA glycosylation and IBD in this study with IgA and IgG glycosylation associated with IBD, IgA Nephropathy, RA and pregnancy associated changes in the recent literature. A = Antennarity, B = Bisection, G = galactosylation, GN = GalNAc, S= Sialylation. ↑ = Trait elevated in cases vs HC, ↓ = Trait lower in cases vs HC. In this study, the associations found between IgA glycosylation and IBD are marked in black. In the other studies, the color green indicates a significant association in the same direction (cases vs HC) as reported here, red indicates association in the opposite direction, and blue indicates reported associations that were not found in our study. The studies used for comparison are part of the bibliography cited in this article.

With our newly developed LC–MS/MS method, we could show that CD differed from HC in terms of IgA1 *O*-glycosylation as well as IgA1 and 2 *N*-glycosylation with regard to antennarity, bisection, galactosylation, and sialylation. In UC, mostly the *O*-glycosylation was altered compared to HC. Overall, for all associations of IgA glycosylation we found between IBD and HC, CD patients seemed to have the more extreme profile of inflammation-associated IgA glycosylation and UC is showing less extreme profiles, similar to what has been found for the total plasma *N*-glycome and in an IgG-specific study.^3,4^ In the following, IgA glycosylation signatures of CD and UC will be discussed in the background of other cohort studies covering IgA glycosylation (Figure 8).

It is possible that with our analytical approach, the changes in glycosylation observed in the IgA captured from serum reflect more global changes in glycosylation of the immune system than what could be observed in localized tissue samples of the inflamed areas. However, sampling serum from patients is far less invasive than sampling tissue and is, thus, a more easily accessible biomarker specimen.

### Antennarity and Bisection

On the LAGY peptide comprising Asn_340_, the glycosylation showed lower antennarity and elevated bisection in CD patients as compared to controls, parallel to what has been observed in other diseases such as RA and IgA nephropathy.^14,15^ In contrast, pregnancy has been found to be associated with lower bisection as well as with a concomitant improvement of the inflammation status in pregnant RA patients.^34^ In other studies, a decrease of antennarity has been observed after delivery for both IgA and IgG.^14,34^ These two glycosylation traits were found to differ significantly between CD and UC, however, the functional role of these differences is not clear. These differences are also mainly IgA1 specific, as IgA2 does not normally contain the LAGC/LAGY glycopeptide but rather has a slightly different protein backbone MAGC/MAGY (in IgA2 isoform #P01877-1) that was not detected in our study. There could be an exception to this in the case of a rare mutation of the IgA2Met_319_ that causes it to have to the same glycopeptide LAG as IgA1.

### Galactosylation

In this study, a lower galactosylation in CD is reported on two glycosylation sites (TPL and LSL, Table S1), while a previous study reported no difference in IgA galactosylation in IBD vs HC.^22^ This might be due to the increased performance of our newly developed analytical workflow, enabling us to handle the larger number of cases analyzed here. Multiple studies have reported lower IgG galactosylation in IBD patients and other diseases as nonspecific inflammation marker and this appears to be in line with our findings for IgA, although with a less prominent effect size.^3,22,35^ It is known that the clearance of IgA can be affected by its terminal *N*-glycosylation motifs and could, thus, be a factor of inflammation in IBD as well.^36,37^ The changes in galactosylation on TPL are IgA2-specific, while the changes observed on LSL are shared between IgA1 and IgA2, which could indicate a similar glycosylation mechanism for both sites and a more pronounced pro-inflammatory profile of IgA2.^23^

### Sialylation

The lower sialylation of *N*-glycans that was found on LSL and TPL glycopeptides of CD patients has also been found to be associated with other diseases such as IgA nephropathy and RA and could be promoting the inflammation and, as discussed for galactosylation, could expose more galactose-terminated glycans and impact IgA clearance from the circulation.^14,15,23^ The opposite effect has been observed during the late stage of pregnancy where elevated sialylation of LSL and LAGY/C glycopeptides were found before a return to normal levels postdelivery, suggesting an anti-inflammatory effect of higher sialylated *N*-glycans of IgA.^13,14^

### *O*-Glycosylation

Overall, the *O*-glycopeptides covered a wide range of compositions (H_3_–_5_N_3_–_6_S_1–5_) containing up to 6 N, e.g., H4N6S2 and H4N6S2, which is in accordance with the literature data that indicate a variation in site occupancy from 3 to 6 sites with predominantly core 1 structures.^31^ Our findings of lower number of GalNAcs on the IgA1 hinge-region *O*-glycopeptide of IBD patients are linked to increased disease activity and are in accordance with previous findings in IBD patients.^22^ These results are also comparable to those found in IgA nephropathy and in RA (Figure 8).^22,38–40^ The similarities of deficient *O*-glycosylation in IBD and other inflammatory diseases indicate a possible role in modulating IgA function that is, however, largely unexplored.^41–45^ An exception to that would be the role of agalactosylated IgA1 hinge region *O*-glycans which has been reported to be a target of autoantibodies in IgA nephropathy.^46^

### Intervention and Medication

The low number of significant associations found between IgA and treatments, whether surgical or medical, is most likely due to the small effect size of the tested associations and the low statistical power possible in this study with the low number of patients in each category (Table 1) with the stringent Bonferroni correction with 270 tests performed (Table S9).

It is interesting to note that the significant traits of IgA glycosylation associated with medication found in this study are similar to what was previously reported with the analysis of the TPNG where lower galactosylation in UC patients was associated with the use of more potent drugs.^4^ In a comparable study about IgAN, patients had lower levels of sialylation on the TPL and LSL glycopeptide compared to the controls.^15^ Here, UC patients who were administered the more potent drugs similarly exhibited lower levels of sialylation on these two glycopeptide clusters.

### Methodological Considerations

While sialic acids usually have a strong effect on the retention time of glycopeptides in reverse-phase LC, as shown here for the MS2 identification data using a gradient containing formic acid, in our high resolution MS1 method used for the high-throughput measurements, we optimized the LC conditions by adding TFA to the eluents to prevent this effect and to allow robust data processing as previously described.^15,47^

For the samples analyzed here, IgA concentration was not measured but it has previously been reported that IgA concentrations do not differ between IBD and HC.^48^ The approach used for the sample preparation included an excess of immunoaffinity beads for capturing, and total area normalization of the extracted glycans during data analysis enabled us to look at changes in the glycosylation profile of IgA without correction for IgA concentration.

The LC–MS method used here is not able to provide absolute quantitative results as no internal standard were included. However, when comparing different disease groups on the same analytes, it is possible to estimate if a cluster, e.g., TPL (which is specific to IgA2), is elevated compared to the other groups. In this regard, as CD had a higher relative abundance of the TPL cluster (data not shown) compared to HC and UC, this could indicate that the ratio IgA1/IgA2 is decreased in CD, and that the difference in ratio could be associated with autoimmune diseases.^23^ This could be further investigated by quantitative assays, such as isotype-specific ELISAs or by adding isotopically labeled standards to the samples for MS analysis.

### Similar Studies

All the associations reported in this study were similarly reported for IgA and IgG in IBD, IgA nephropathy, and other autoimmune disease like RA, except for the sialylation of the *O*-glycans in RA and the bisection of IgG in CD vs HC (Figure 8).^3,13–15,22,34,38,40,45,49^ The changes in IgA glycosylation with IBD were mostly opposite of the associations reported during pregnancy, similar to IgG where glycosylation changes with inflammatory conditions were opposed to those occurring with pregnancy.^13,14,34^

## Conclusions

In this study, we applied our recently developed IgA glycopeptide analytical workflow to a large number of samples and reported novel associations of IgA glycosylation with IBD. Our prediction model had a good performance to predict CD versus HC while the discrimination power for UC was lower. The possibility to detect IBD and discriminate CD from UC should be considered with using readily available biofluids as a first tentative approach for the early diagnosis of IBD before attempting invasive examination procedures for the comfort of the patients.

## Supporting Information Available

Properties of analytes of IgA glycopeptides; byonic peptide fragmentation and identification; derived traits calculations; associations of IgA glycopeptides with age and sex; associations of IgA glycopeptides with IBD; associations of IgA glycopeptides with disease location in CD; associations of IgA glycopeptides with disease behavior in CD; associations of IgA glycopeptides with disease location in UC; associations of IgA glycopeptides with surgery; associations of IgA glycopeptides with drug strength; byonic identification of the glycopeptides TPL; byonic identification of the glycopeptides LAGC/Y; byonic identification of the glycopeptides LSL; byonic identification of the glycopeptides HYT; glycopeptide clusters and MS1 annotation of TPL; glycopeptide clusters and MS1 annotation of LAGC; glycopeptide clusters and MS1 annotation of LAGY; glycopeptide clusters and MS1 annotation of LSL; and glycopeptide clusters and MS1 annotation of HYT (XLSX)
Byonic identification of the glycopeptides TPL; byonic identification of the glycopeptides LAGC/Y; byonic identification of the glycopeptides LSL; byonic identification of the glycopeptides HYT; glycopeptide clusters and MS1 annotation of TPL; glycopeptide clusters and MS1 annotation of LAGC; glycopeptide clusters and MS1 annotation of LAGY; glycopeptide clusters and MS1 annotation of LSL; glycopeptide clusters and MS1 annotation of HYT (PDF)

## Notes

Raw Data: Mass spectrometry proteomics data raw mzXML datafiles have been deposited to the CMMS MassIVE archive [doi:10.25345/C5W08WS34] [data set license: CC0 1.0 Universal (CC0 1.0)] at ftp://MSV000091731@massive.ucsd.edu (FTP download link) and ftp://massive.ucsd.edu/MSV000091731/ (public URL).
