# Supplementary material for: Immunoglobulin A Glycosylation Differs between Crohn’s Disease and Ulcerative Colitis
Source: J Proteome Res. 2023 Aug 29;22(10):3213–24. doi: 10.1021/acs.jproteome.3c00260 (PMC10563165; doi:10.1021/acs.jproteome.3c00260)

Supplementary Figures for the manuscript Immunoglobulin A glycosylation differs between Crohn's Disease and Ulcerative Colitis

Florent Clerc[1], Karli R. Reiding[1,2,3], Noortje de Haan[1], Carolien A. M. Koeleman [1], Agnes L. Hipgrave Ederveen[1], Natalia Manetti [4,5], IBD-BIOM consortium, Viktoria Dotz [1#], Vito Annese [4,6,7], Manfred Wuhrer[1*]

[1]*Center for Proteomics and Metabolomics, Leiden University Medical Center (LUMC), Postbus 9600, 2300 RC Leiden, The Netherlands*

[2]*Biomolecular Mass Spectrometry and Proteomics, Bijvoet Center for Biomolecular Research and Utrecht Institute for Pharmaceutical Sciences, University of Utrecht, Padualaan 8, 3584 CH Utrecht, The Netherlands*

[3]*Netherlands Proteomics Center, Padualaan 8, 3584 CH Utrecht, The Netherlands.*

[4]*Unit of Gastroenterology SOD2 (Strutture Organizzative Dipartimentali), Azienda Ospedaliero Universitaria (AOU) Careggi, Florence, Italy.*

[5]*Gastroenterology Unit, San Jacopo Hospital, Pistoia, Italy*

[6]*Unit of Gastroenterology, IRCCS (Istituto di Ricovero e Cura a Carattere Scientifico–Casa Sollievo della Sofferenza) Hospital, San Giovanni Rotondo, Italy.*

[7]*Vita-Salute San Raffaele University Faculty of Medicine and Surgery, Milano, Italy*[7]*IRCCS Policlinico San Danato, San Donato Milanese, Italy*

[#]Current address: *BioTherapeutics Analytical Development, Janssen Biologics BV, Einsteinweg 101, 2333 CB Leiden, The Netherlands*

*Corresponding Author
e-mail: m.wuhrer@lumc.nl, Phone: +31-71-5266989.

Supplementary figures

SF1:Byonic identification of the glycopeptides TPL
SF2:Byonic identification of the glycopeptides LAGC/Y
SF3:Byonic identification of the glycopeptides LSL
SF4:Byonic identification of the glycopeptides HYT
SF5: Glycopeptide clusters and MS1 annotation of TPL
SF6: Glycopeptide clusters and MS1 annotation of LAGC
SF7: Glycopeptide clusters and MS1 annotation of LAGY
SF8: Glycopeptide clusters and MS1 annotation of LSL
SF9: Glycopeptide clusters and MS1 annotation of HYT

SF1:Byonic identification of the glycopeptides TPL

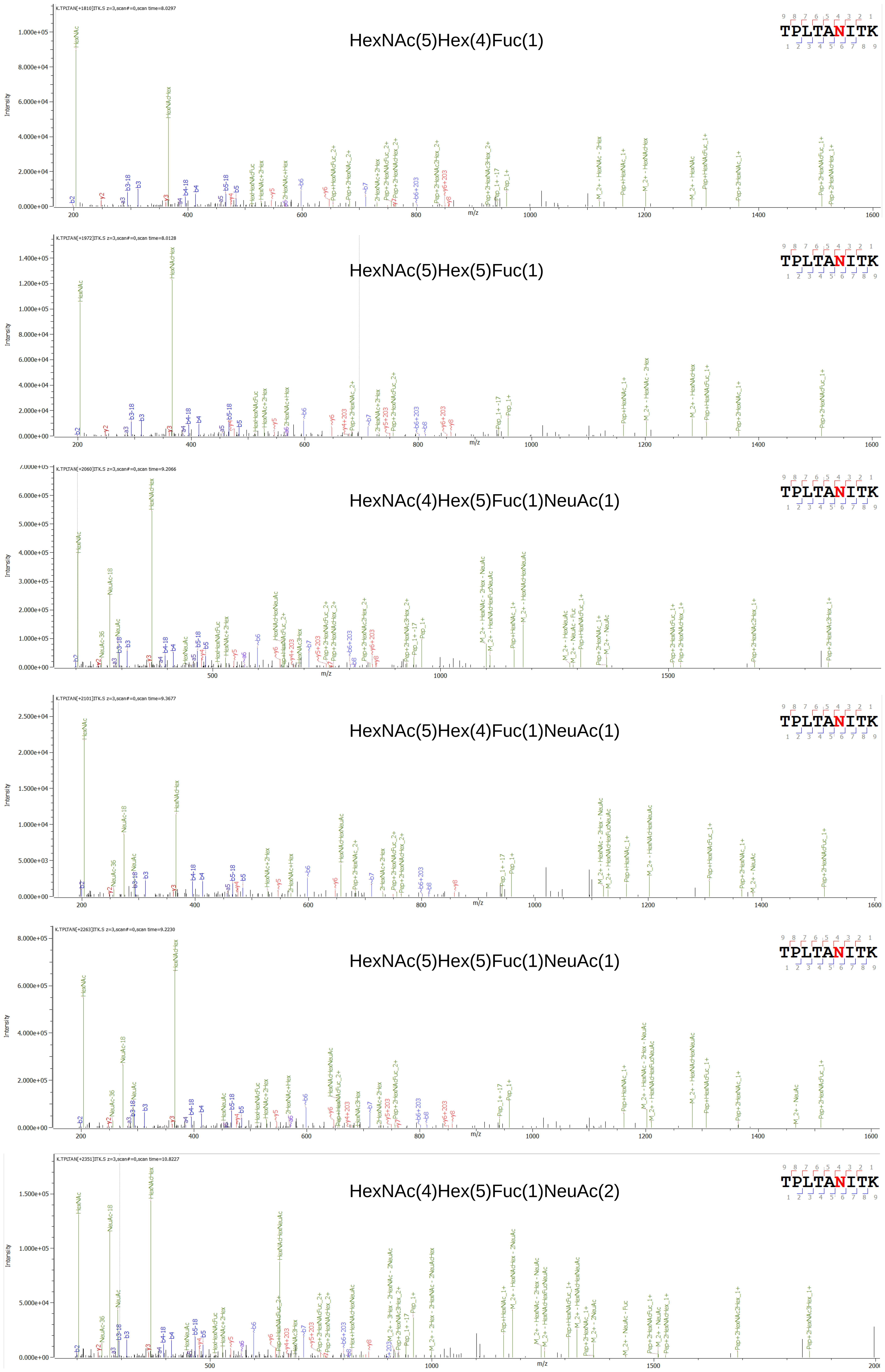

SF2:Byonic identification of the glycopeptides LAGC/Y

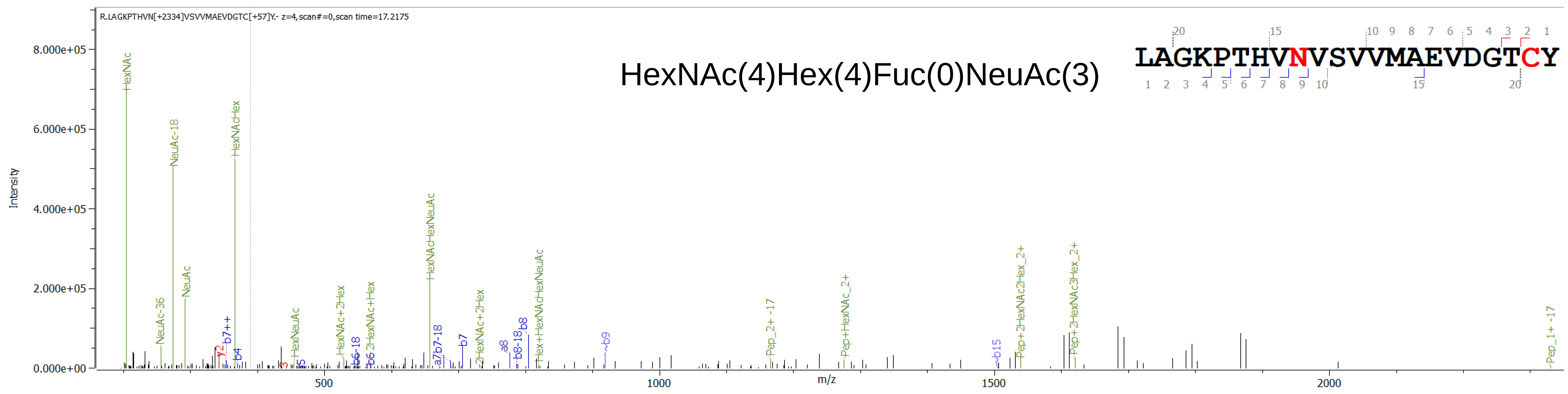

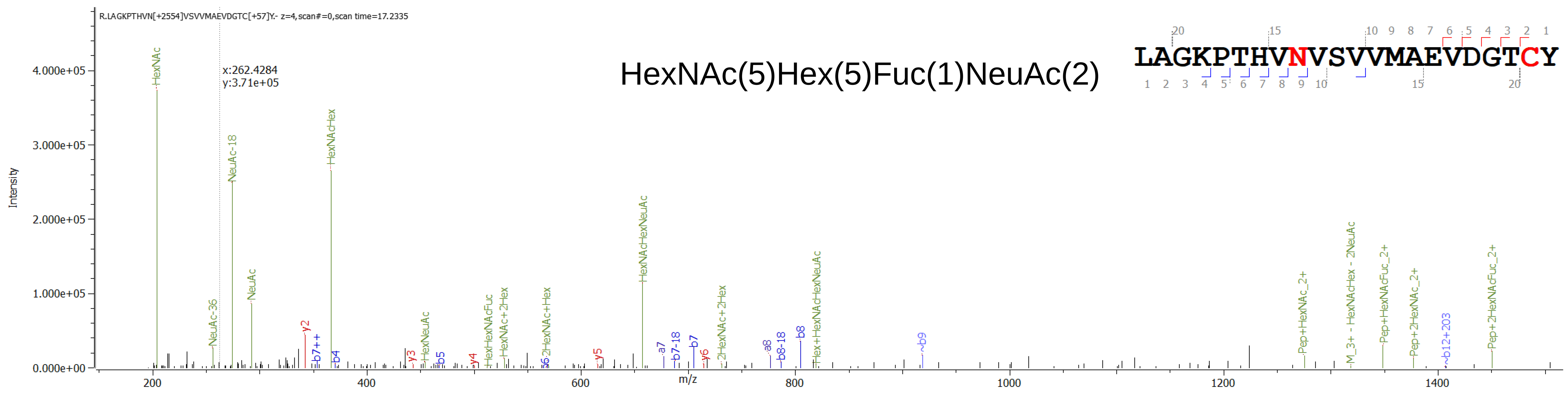

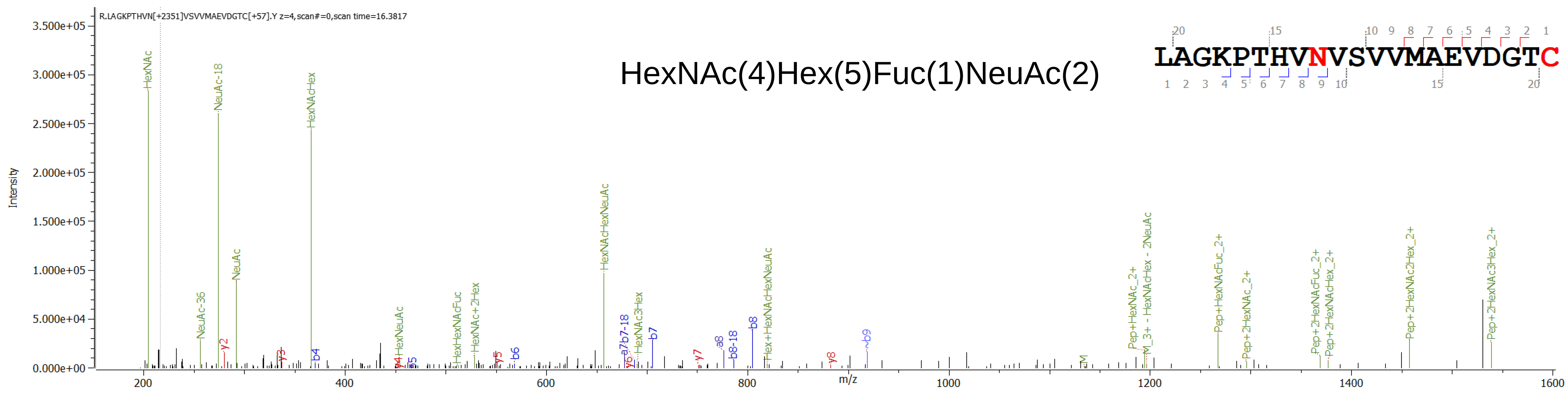

SF3:Byonic identification of the glycopeptides LSL

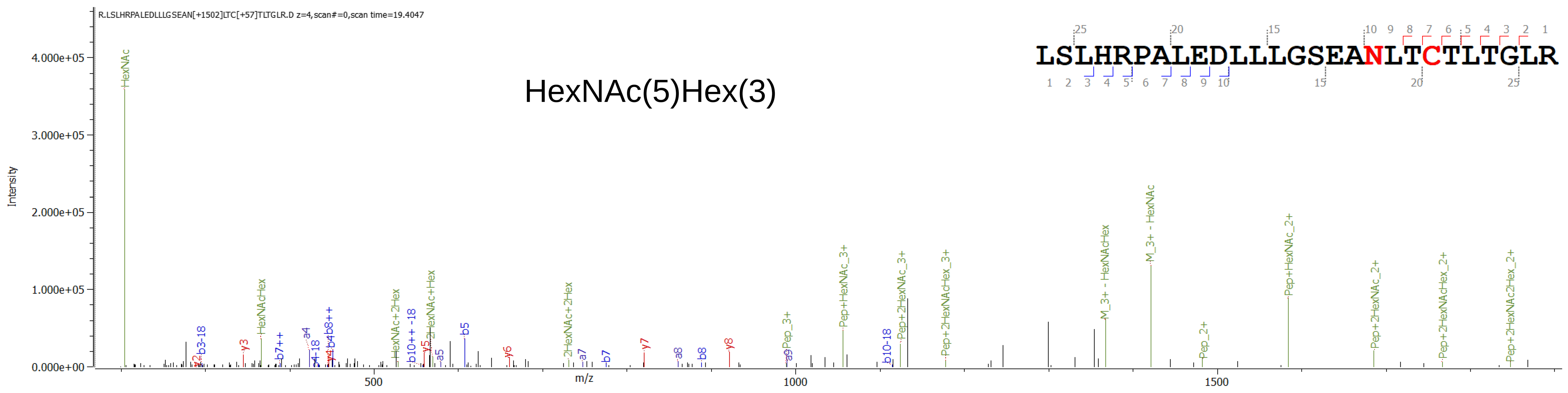

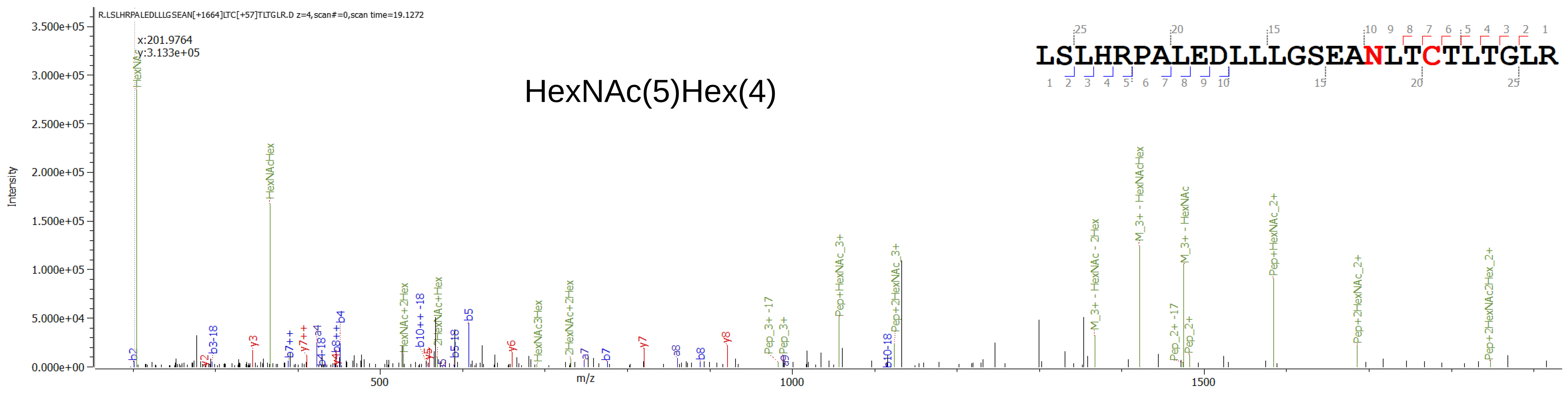

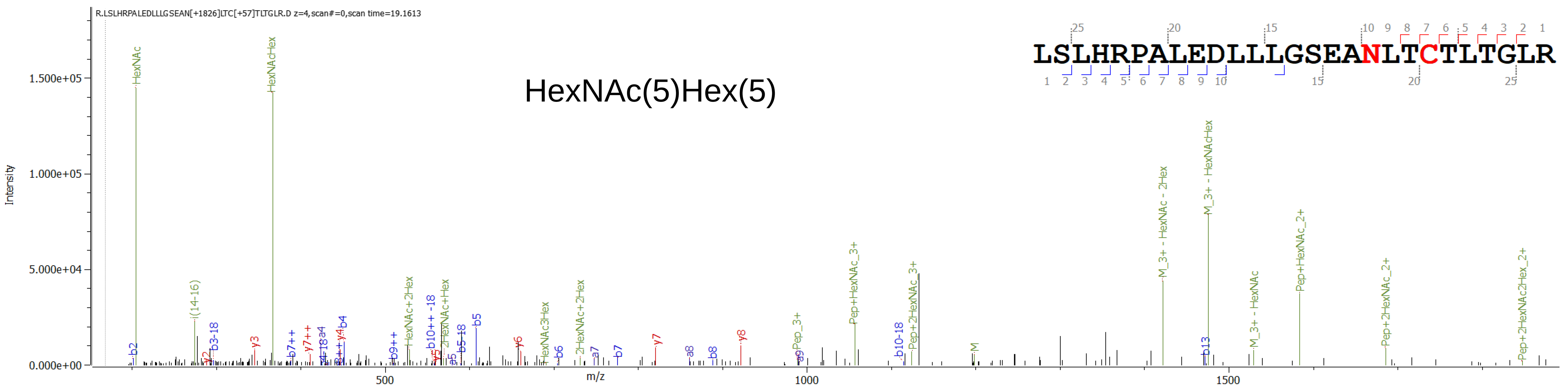

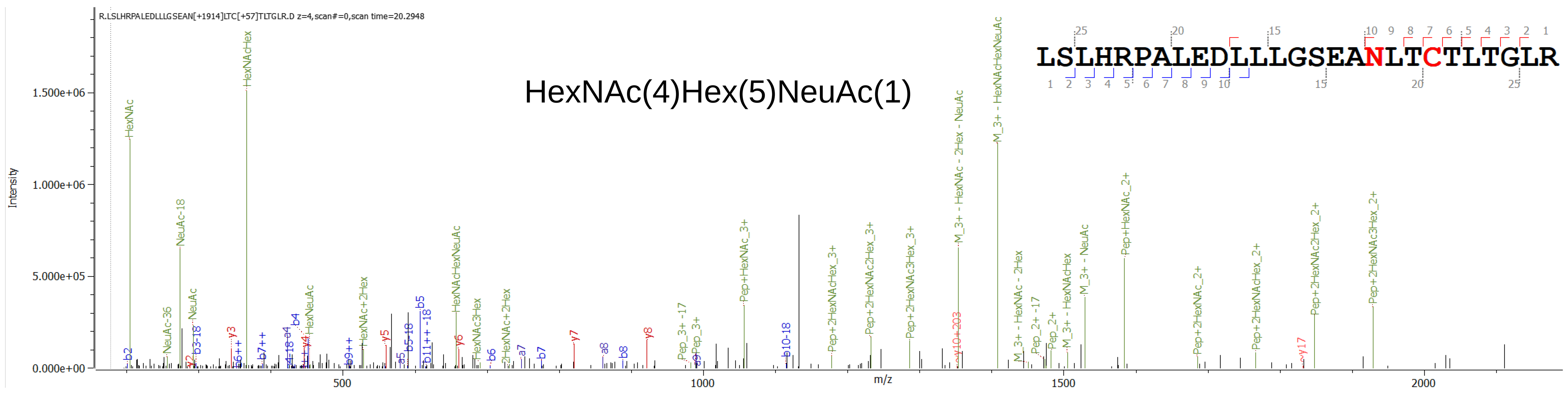

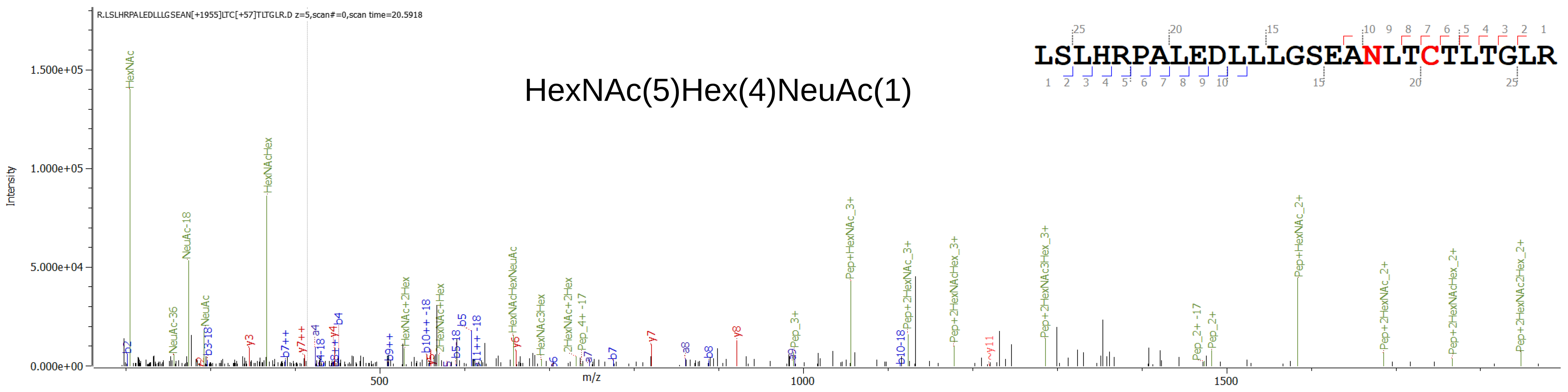

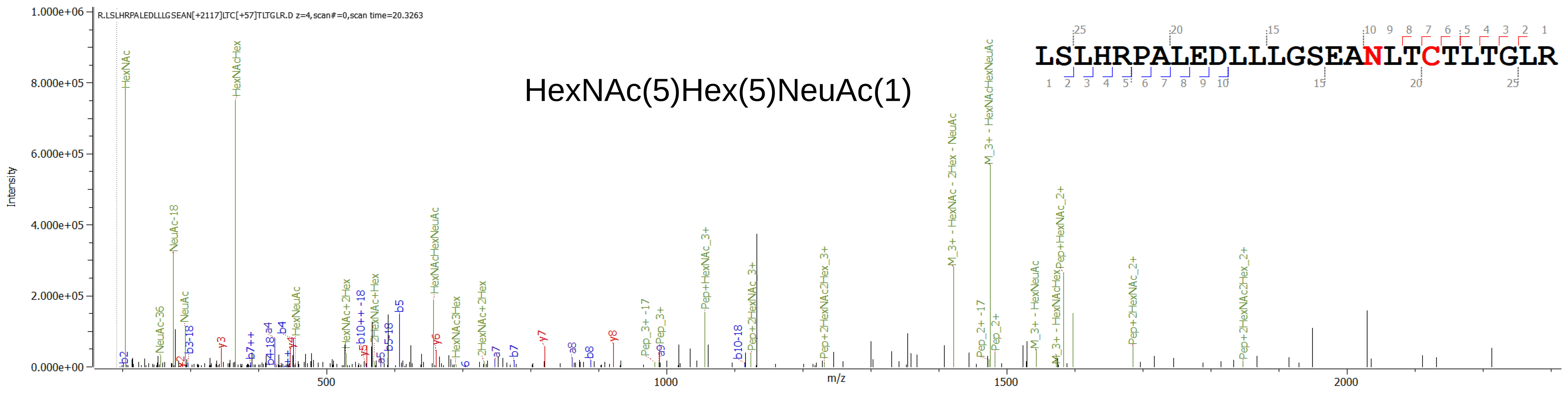

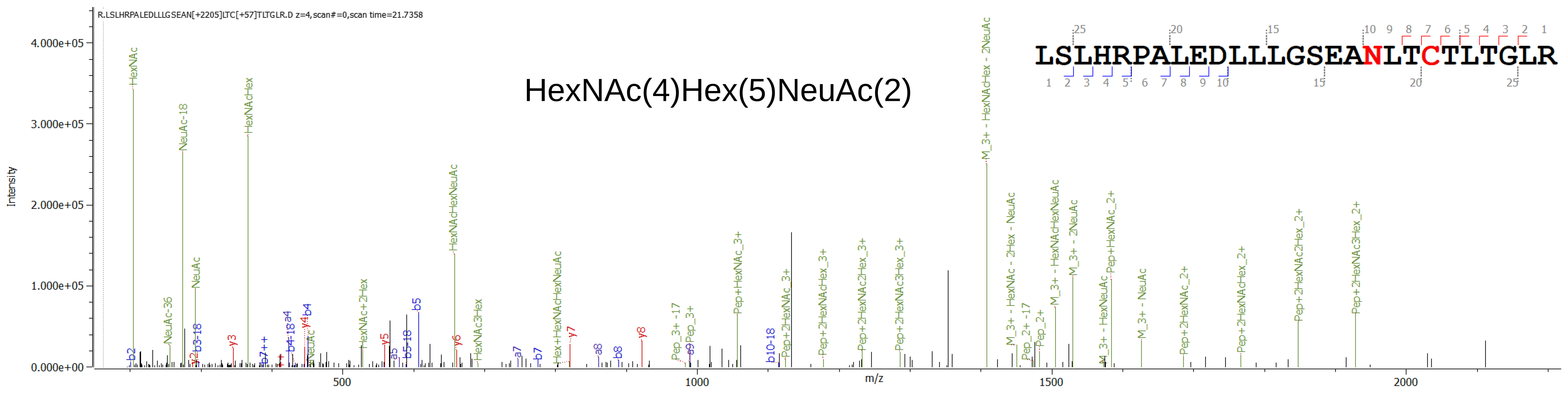

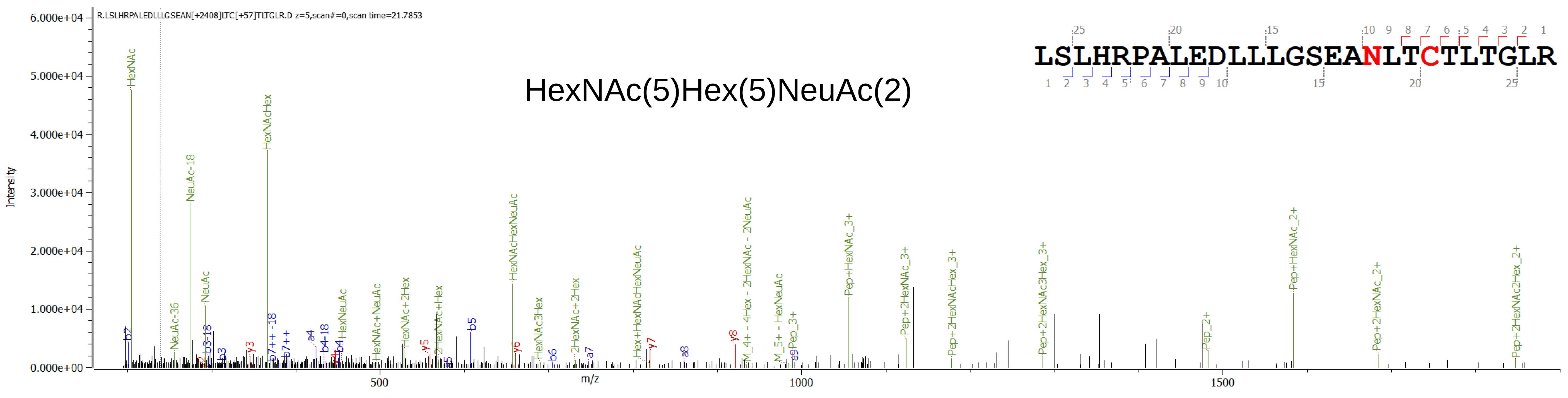

SF4:Byonic identification of the glycopeptides HYT

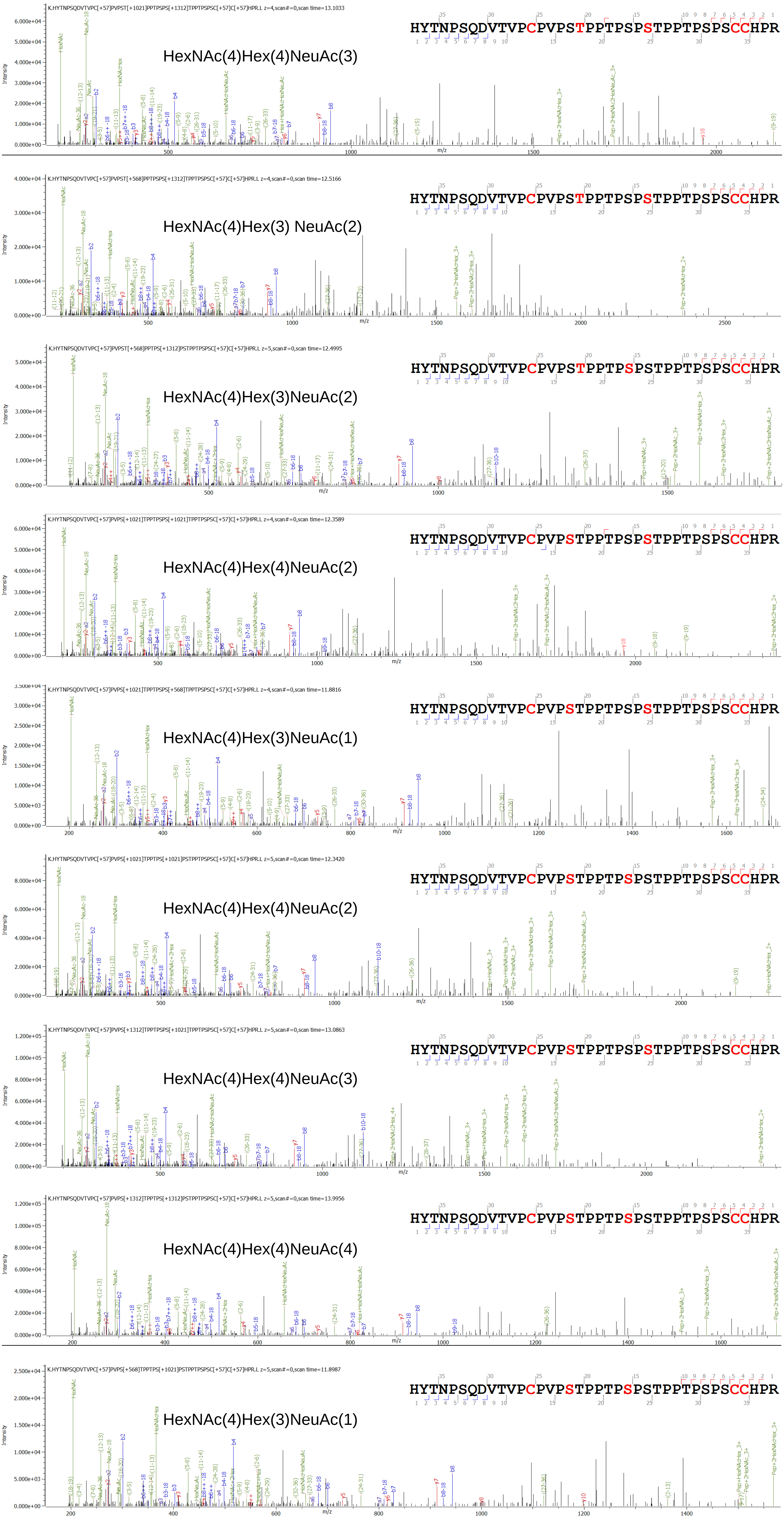

SF5: Glycopeptide clusters and MS1 annotation of TPL

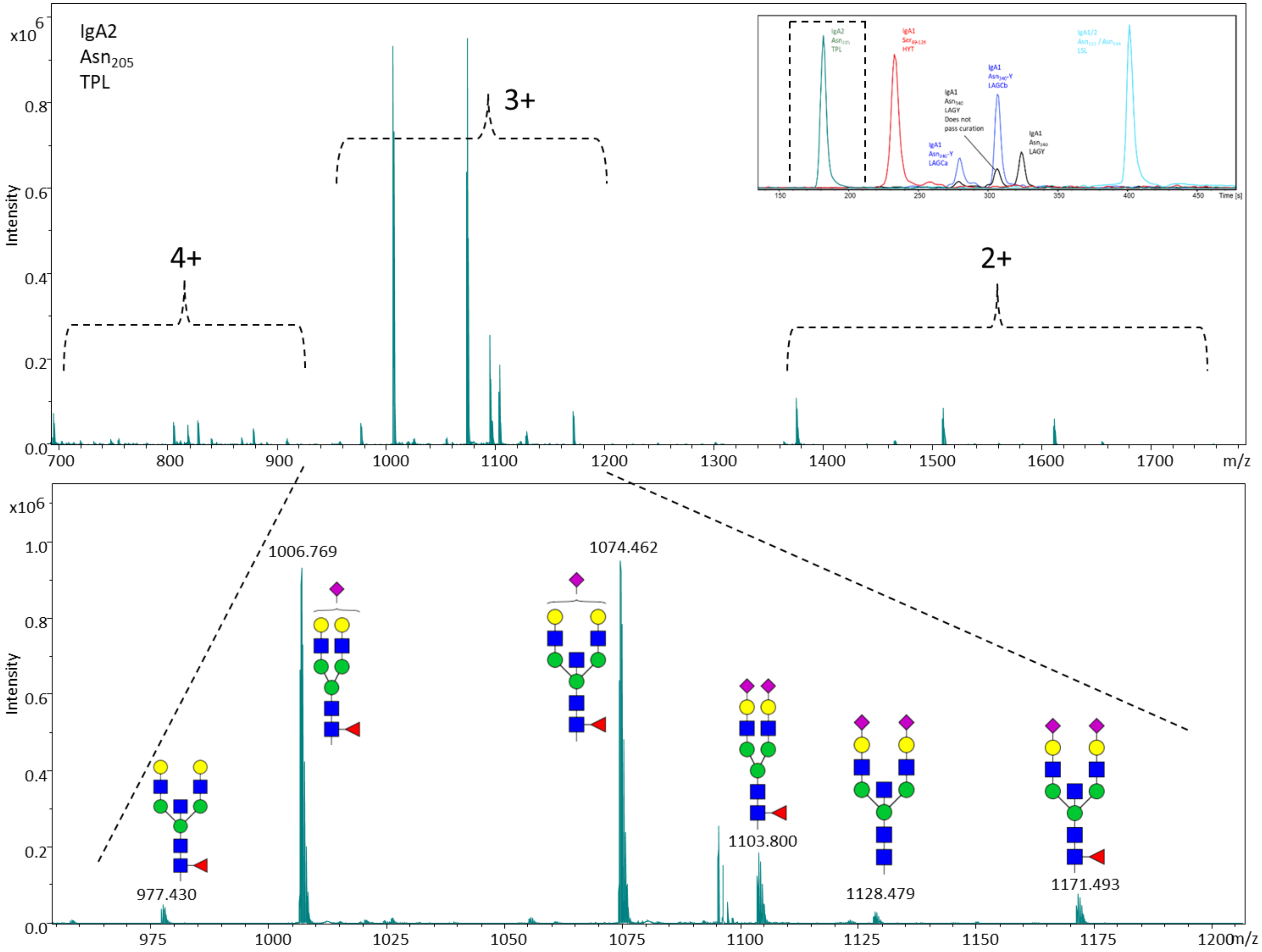

SF6: Glycopeptide clusters and MS1 annotation of LAGC

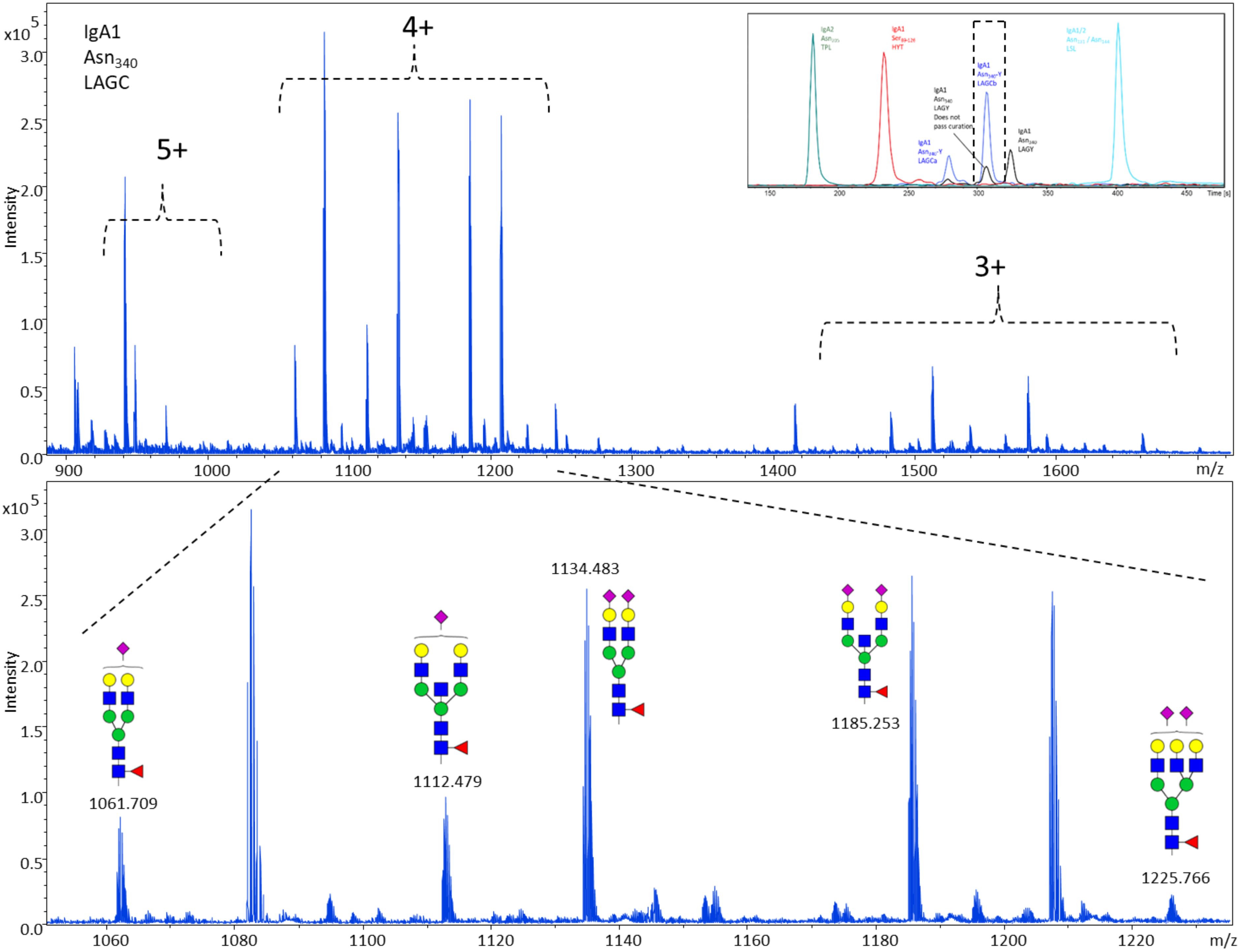

SF7: Glycopeptide clusters and MS1 annotation of LAGY

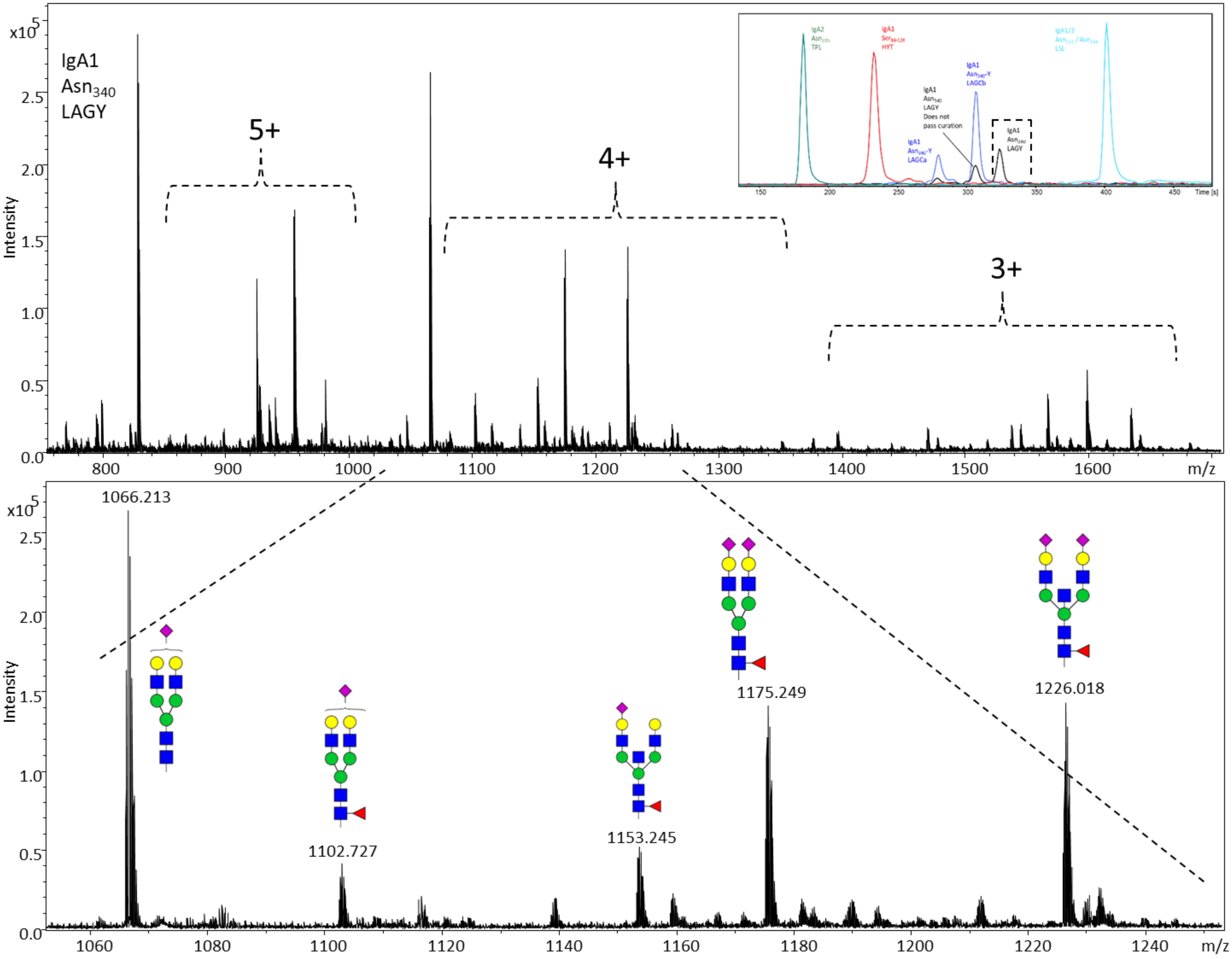

SF8: Glycopeptide clusters and MS1 annotation of LSL

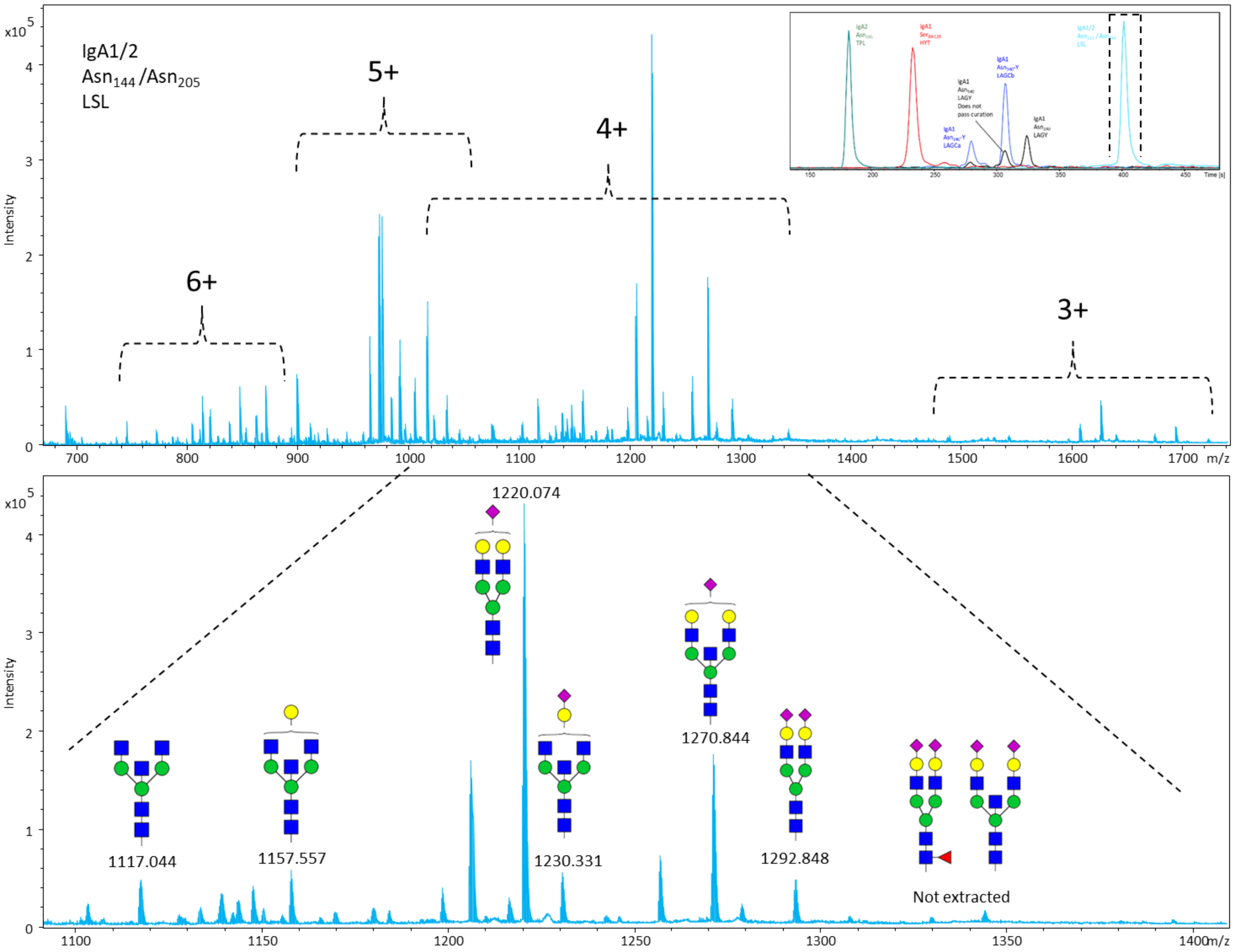

SF9: Glycopeptide clusters and MS1 annotation of HYT

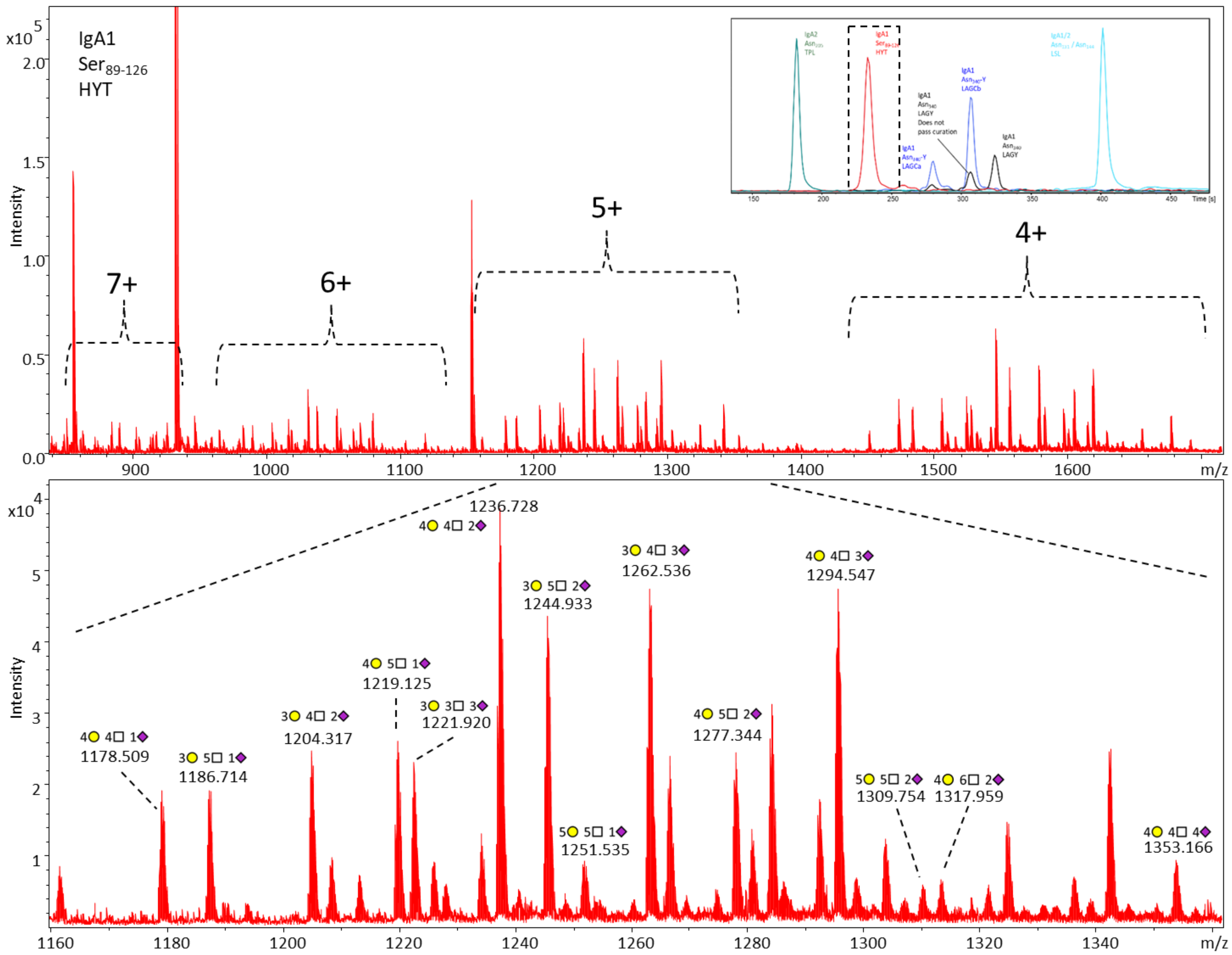

Supplement: Supplementary file 2 — pr3c00260_si_003.pdf [file pr3c00260_si_003.pdf]
